# Synthesis and Structure‐Activity Relationships of *N*‐(4‐Benzamidino)‐Oxazolidinones: Potent and Selective Inhibitors of Kallikrein‐Related Peptidase 6

**DOI:** 10.1002/cmdc.201900536

**Published:** 2019-11-18

**Authors:** Elena De Vita, Niels Smits, Helma van den Hurk, Elizabeth M. Beck, Joanne Hewitt, Gemma Baillie, Emily Russell, Andrew Pannifer, Véronique Hamon, Angus Morrison, Stuart P. McElroy, Philip Jones, Natalia A. Ignatenko, Nikolas Gunkel, Aubry K. Miller

**Affiliations:** ^1^ Cancer Drug Development Group German Cancer Research Center (DKFZ) Im Neuenheimer Feld 280 69120 Heidelberg Germany; ^2^ Faculty of Biosciences University of Heidelberg 69120 Heidelberg Germany; ^3^ Pivot Park Screening Centre Kloosterstraat 9 5349 AB Oss (The Netherlands; ^4^ European Screening Centre Newhouse (ESC) Biocity Scotland Bo'ness Road ML15UH Newhouse Scotland; ^5^ University of Arizona Cancer Center University of Arizona Tucson AZ 85721 USA; ^6^ Department of Cellular and Molecular Medicine University of Arizona Tucson AZ 85721 USA; ^7^ German Cancer Consortium (DKTK) 69120 Heidelberg Germany

**Keywords:** Drug Discovery, High-throughput screening, Medicinal Chemistry, Structure-activity relationship, Protease inhibitors

## Abstract

Kallikrein‐related peptidase 6 (KLK6) is a secreted serine protease that belongs to the family of tissue kallikreins. Aberrant expression of KLK6 has been found in different cancers and neurodegenerative diseases, and KLK6 is currently studied as a potential target in these pathologies. We report a novel series of KLK6 inhibitors discovered in a high‐throughput screen within the European Lead Factory program. Structure‐guided design based on docking studies enabled rapid progression of a hit cluster to inhibitors with improved potency, selectivity and pharmacokinetic properties. In particular, inhibitors **32** ((5*R*)‐3‐(4‐carbamimidoylphenyl)‐*N*‐((*S*)‐1‐(naphthalen‐1‐yl)propyl)‐2‐oxooxazolidine‐5‐carboxamide) and **34** ((5*R*)‐3‐(6‐carbamimidoylpyridin‐3‐yl)‐*N*‐((1*S*)‐1‐(naphthalen‐1‐yl)propyl)‐2‐oxooxazolidine‐5‐carboxamide) have single‐digit nanomolar potency against KLK6, with over 25‐fold and 100‐fold selectivities against the closely related enzyme trypsin, respectively. The most potent compound, **32**, effectively reduces KLK6‐dependent invasion of HCT116 cells. The high potency in combination with good solubility and low clearance of **32** make it a good chemical probe for KLK6 target validation in vitro and potentially in vivo.

## Graphical abstract

**A successful screen** performed within the European Lead Factory program, followed by docking studies, has resulted in compound **32**, a potent and selective *N*‐(4‐benzamidino)‐oxazolidinone inhibitor of kallikrein‐related peptidase 6 (KLK6). Aberrant levels of this serine protease have been found in different malignancies and in neurodegenerative diseases. Good solubility and low clearance of **32** make it a good probe for KLK6 target validation.

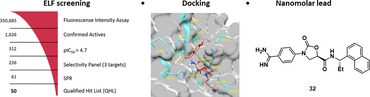

## Introduction

Kallikrein‐related peptidase 6 (KLK6), previously known as protease M, zyme, neurosin, or myelencephalon specific protease,^1^ is a secreted serine protease that belongs to the family of tissue kallikreins (KLKs).^2^ Like all 15 KLKs, KLK6 is released into the extracellular matrix as a zymogen and activated upon cleavage of a pro‐peptide, a process which can be mediated by other proteases such as KLK5,^3^ plasmin,^4^ urokinase (uPA),^4^ and MMP‐20.^5^ Removal of the pro‐peptide generates mature KLK6, a trypsin‐like enzyme with cleavage specificity after basic P1 residues, preferably arginine. Broader sequence requirements have been reported for the flanking residues (P2, P3, P1′, and P2′).^6^ Relevant endogenous substrates of KLK6 have been identified in vitro and include protease‐activated receptors (PARs),^7^ α‐synuclein,^7^, ^8^ and myelin basic protein.^9^

Secreted proteases (e. g. KLKs and matrix‐metalloproteinases) are investigated as potential therapeutic drug targets due to their role in extracellular signaling via proteolysis‐mediated production of small signaling molecules or proteolytic activation of membrane receptors.^10^ KLK6 can activate PARs, and this signaling pathway has been found to be dysregulated in cutaneous malignant melanoma.^11^ In this cancer, KLK6 was found to be secreted by the keratinocytes surrounding the tumor cells in response to stimuli from the tumor, and to act in a paracrine fashion to activate PAR‐1 receptors, which are overexpressed on melanocytes. This signaling cascade was found to have an effect on tumor migration and invasiveness in vitro^11^ and is considered to contribute to recurrence and metastasis in melanoma patients that undergo surgery.^12^ KLK6‐promoted migration was also observed in colon cancer, where knockdown of KLK6 reduced migration and invasion of HCT116 cells in vitro.^13^ Furthermore, in an orthotopic colon cancer mouse model, mice injected with KLK6 positive HCT116 cells had significantly more metastases and worse survival than mice injected with shKLK6 HCT116 clones.^13^ Nevertheless, the role of KLK6 needs further investigation in these and other types of cancers, as its role is clearly tumor‐dependent. In head‐and‐neck cancer for example, high levels of KLK6 have been associated with a better prognosis for the patients, resulting in reduced aggressiveness of the disease.^14^

In addition to malignancies, KLK6 is studied in the central nervous system (CNS), where its physiological abundance might imply an important role for KLK6 in the maintenance of homeostasis in these organs. In neurodegenerative diseases such as multiple sclerosis, Alzheimer's^15^ and Parkinson's,^16^ as well as spinal cord injury,^17^ aberrant levels of KLK6 have been reported. Potential therapeutic approaches targeting KLK6 have been investigated but require further validation, particularly with high‐quality KLK6 chemical probes.^9^

**Figure 1:**
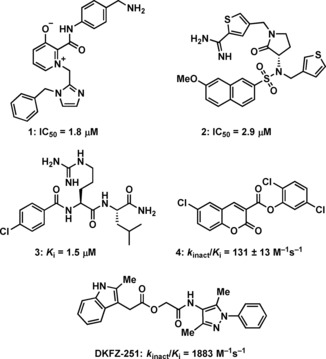
Published KLK6 inhibitors.

To date, few accounts of reversible KLK6 inhibitors have been reported, the most relevant being two sets of small molecules (e. g. **1** and **2**, Figure 1) discovered by in silico high‐throughput screening (HTS) supported by X‐ray crystallography,^18^ and a series of pseudopeptides (e. g. **3**), which were reported to be highly selective over the closely related KLK5.^19^ Covalent coumarin‐based suicide inhibitors (e. g. **4**)^20^ as well as transient quiescent affinity labelers (e. g. **DKFZ**‐**251**), our first disclosure of KLK6 inhibitors,^21^ have also been reported.

Given the growing interest in KLK6 as a drug target and the potential benefit of being able to control its enzymatic activity to validate current biological hypotheses, we set out to find a novel series of selective reversible KLK6 inhibitors.

## Results and Discussion

### High‐Throughput Screening and Validation of a Promising Hit

**Figure 2:**
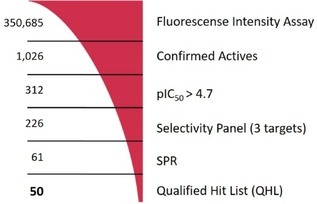
Visualization of the HTS triage process to generate the QHL.

Using our previously published KLK6 assay format,^21^ we performed a HTS with the European Lead Factory (ELF),^22^ and tested ∼350,000 substances at 10 μM for their ability to reduce KLK6‐catalyzed hydrolysis of the fluorogenic Boc‐Phe‐Ser‐Arg‐AMC peptide. About 8,000 actives were identified and re‐tested. After applying a threshold cutoff of 25 % inhibition, and discarding compounds with inherent high fluorescence (>3 times the background), 1026 compounds were selected for dose‐response curve analysis (Figure 2). These substances were tested at seven concentrations from 20 μM to 20 nM, resulting in 312 entities with a pIC_50_>4.7. A selectivity screen against trypsin, thrombin and factor Xa, as previously reported,^21^ reduced the number to 226 preliminary hits. Further validation of these hits was performed via surface plasmon resonance (SPR), resulting in 61 likely reversible KLK6 binders. LC‐MS analysis eliminated 4 impure substances, and a qualified hit list (QHL) of 50 compounds was generated. These were sorted into ten structural clusters with fifteen singletons. Many of the hits contained a highly basic moiety such as amidine or guanidine, functional groups that are often found in trypsin‐like serine protease inhibitors and sometimes associated with poor cellular permeability. Nevertheless, we chose to further advance with such compounds because KLK6 is a secreted protease, mindful that permeability might need to be considered at an early stage.

Compounds containing an oxazolidinone benzamidine were the largest and most promising cluster with nine members. All of the compounds in the cluster were potent enough to generate a pIC_50_ in the primary dose‐response assay (Table 1). Each substance also gave p*K*_D_ values in the SPR assay which were similar to the pIC_50_ values. Furthermore, hits from this cluster exhibited on/off binding kinetics in the SPR assay consistent with a reversible mechanism of action (data not shown). In addition, hints of structure‐activity relationships (SAR) could be gleaned. For example, the most potent hit (**5**) is the only compound in the cluster with an amide directly linked to the oxazolidinone. Compounds with an amide extended by one carbon (**6**) or containing other functional groups (**7** and **8**) are significantly less active. In addition, **5** had by far the best selectivity profile relative to trypsin, thrombin and factor Xa (vide infra).

**Table 1:** Four members of the top hit cluster.

Cmpd	Structure	KLK6	
		pIC_50_^[a]^	p*K*_D_^[b]^
**5**	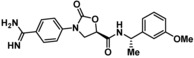	7.2	7.0
**6**	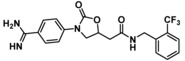	5.0	4.9
**7**	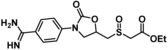	5.8	4.7
**8**	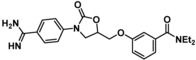	5.1	5.1

[a] pIC_50_ measured in the enzymatic inhibition assay. [b] p*K*_D_ measured in the SPR binding assay.

### Determination of the Active Stereochemical Series

Hit **5** was resynthesized along with all three of its stereoisomers **9**–**11**. The activity of **5** against KLK6 was confirmed, although a consistently lower pIC_50_ (6.6 vs. 7.2) was found relative to the same substance in the HTS library (Table 2). Encouragingly, the other three stereoisomers showed poor activity against KLK6, suggesting only the (*R,S*) configuration of the scaffold allows for productive molecular interactions between the inhibitor and KLK6.

**Table 2:** Testing of the four stereoisomers of **5** indicate that **5** is the active stereoisomer.

Cmpd	Structure	KLK6 pIC_50_^[a]^
**5**	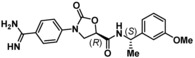	6.6
**9**	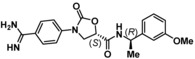	<4.7
**10**	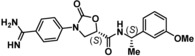	<4.7
**11**	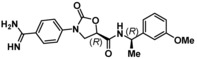	<4.7

[a] pIC_50_ measured in the enzymatic inhibition assay.

### Docking Study Predicts Key Binding Interactions

**Figure 3:**
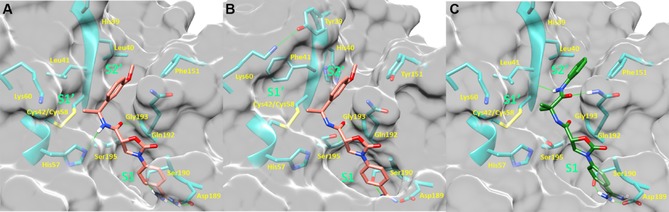
(A) Docking model of **5** (coral) bound to KLK6. (B) Docking model of **5** (coral) overlaid on trypsin (PDB ID 1GJ6). (C) Docking model of **28** (green) bound to KLK6. Both KLK6 models are derived from PDB ID 4D8N.

Compound **5** was docked into an X‐ray crystal structure of KLK6 (PDB ID 4D8N), allowing us to model the main interactions with the target (Figure 3A). As expected, the highest scoring poses predict that the amidine group makes critical hydrogen bonds (H‐bonds) with Asp189 and Ser190 in the S1 pocket and is likely responsible for a significant amount of the binding energy. A secondary H‐bond network is also formed by the amide: the carbonyl of the amide interacts with the backbone of Gly193 in the anionic hole and the amide NH interacts with His57 of the catalytic triad. The more solvent exposed S1′/S2′ regions are occupied by the lipophilic α‐methylbenzyl amine substituent. Close inspection suggested that binding could be increased through more effective filling of the S1′/S2′ pockets lined by Leu40, Leu41, and Phe151. Comparison of our KLK6 model and X‐ray structures of trypsin,^23^ KLK4,^24^ and KLK8^25^ suggested that by exploiting subtle differences between the enzymes’ S1′ and S2′ pockets, we could modulate selectivity for KLK6. For example, the S1′ pocket appears more compact in KLK6 with Lys60, Leu41, and the Cys42/Cys58 disulfide bond forming a tight lipophilic space. In trypsin, the S1′ pocket features Lys60, Phe41 and Cys42/58; however, the Lys60 side chain appears pulled back by an intra‐residue H‐bond with Tyr39, creating a more open pocket which may prefer to accommodate larger groups (Figure 3B). We also predicted that it could be possible to improve binding by adding an additional peptide bond to the inhibitor scaffold, thereby adding an H‐bond to the Leu41 backbone carbonyl and the Gln192 side chain (Figure 3C). On the basis of our modeling, initial medicinal chemistry efforts were invested into increasing affinity toward the S1′/S2′ pockets, which showed the highest potential for exploration and structural expansion. Furthermore, the model was tested by synthesizing and testing substances which lacked specific features that were predicted to be key for binding.

### Synthesis of *N*‐Benzamidine‐Oxazolidinone Derivatives

**Scheme 1:**
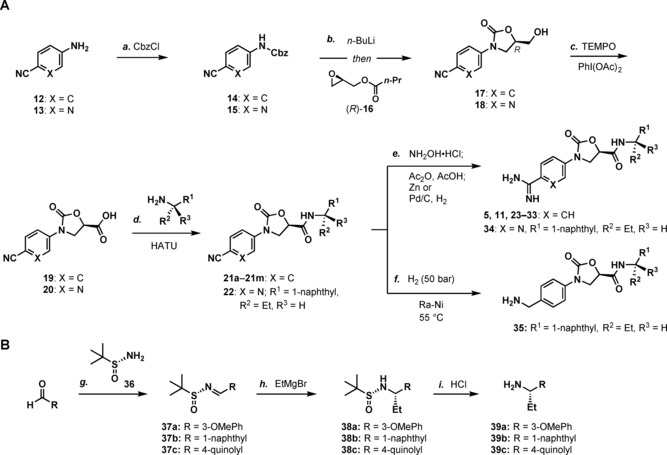
Reagents and conditions: (a) Benzyl chloroformate, Et_3_N, CH_2_Cl_2_; (b) *n*‐BuLi, THF, −78 °C, then **16**; (c) TEMPO, PhI(OAc)_2_, 1 : 1 MeCN/H_2_O; (d) amine, HATU, *i*‐Pr_2_NEt, DMF; (e) NH_2_OH ⋅ HCl, *i*‐Pr_2_NEt, EtOH, 100 °C, μwave, 60 min, then concentrate; Ac_2_O, AcOH, 20 h, then add Zn, 4 h or concentrate then 10 % Pd/C, H_2_ (10 bar), 1 : 1 MeOH/EtOAc; (f) H_2_ (50 bar), Raney‐Ni, EtOH, 55 °C; (g) **36**, 5 mol % PPTS, MgSO_4_ (5 equiv.), CH_2_Cl_2_; (h) EtMgBr, CH_2_Cl_2_, −40 °C; (i) 4 M HCl in dioxane, MeOH.

Compounds for this study were synthesized as depicted in Scheme 1A, beginning with conversion of 4‐aminobenzonitrile (**12**) to the corresponding benzyl carbamate **14**. The key enantiomerically enriched oxazolidinone **17** was prepared by treating commercially available (*R*)‐glycidyl butyrate ((*R*)‐**16**) with the lithium salt of **14**. This cascade transformation begins with opening of the epoxide by the lithio‐carbamate, followed by oxazolidinone ring closure from the resulting alkoxide. Subsequent in situ transesterification of the butyrate ester with benzyl alkoxide provides **17**. Primary alcohol **17** was oxidized in one step to carboxylic acid **19** with TEMPO/PhI(OAc)_2_, and then coupled with various amines to give amides **21a**–**21m**. In some cases, chiral racemic amines were used in the amide coupling step, and the resulting diastereomers were separated by chromatography. To obtain non‐commercial enantioenriched chiral amines **39a**–**39c** for the above amide coupling, we utilized Ellman′s *t*‐butylsulfinamide chemistry (Scheme 1B).^26^ Conversion of the cyano group in **21a**–**21m** to an amidine was performed in a one‐pot, three‐step process: addition of hydroxylamine, yielding an amidoxime, which was acetylated and then reduced with zinc dust or with Pd/C and H_2_ to give **5**, **11**, and **23**–**33**. Compounds **9** and **10** (not depicted in Scheme 1) were synthesized in analogy to **5** and **11**, but using (*S*)‐glycidyl butyrate ((*S*)‐**16**) in the second step. Compound **34** was prepared similarly, starting with 2‐cyano‐5‐aminopyridine (**13**) instead of **12**. Benzyl amine **35** was prepared from the corresponding nitrile via hydrogenation with Raney nickel.

**Scheme 2:**
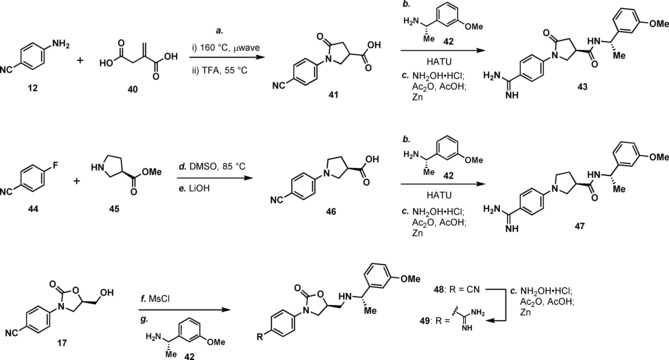
Reagents and conditions: (a) i. neat, 160 °C; ii. TFA, 55 °C; (b) **40**, HATU, *i*‐Pr_2_NEt, DMF; (c). NH_2_OH ⋅ HCl, *i*‐Pr_2_NEt, EtOH, 100 °C, μwave, 60 min, then concentrate; Ac_2_O, AcOH, 20 h then add Zn, 4 h; (d) DMSO, 85 °C; (e) LiOH, H_2_O/THF; (f) methanesulfonyl chloride, Et_3_N, CH_2_Cl_2_; (g) **40**, DMF, 120 °C, μwave.

The synthesis of three additional substances is depicted in Scheme 2. In the first example, **12** and 2‐methylenesuccinic acid (**40**) were combined to make lactam **41** as a racemate, which was advanced to **43** via amide coupling with **42** followed by amidine formation. Amide **43** was separated from the diastereomer arising from the undesired enantiomer contained in *rac*‐**41** via chromatography. The synthesis of **47** began with an S_N_Ar reaction between 4‐fluorobenzonitrile (**44**) and methyl (*R*)‐pyrrolidine‐3‐carboxylate (**45**) to give after saponification pyrrolidine **46**, which was advanced to **47** as above. Compound **49** was synthesized starting with conversion of alcohol **17** to amine **48**, via activation of **17** as a mesylate and displacement with amine **42**. Conversion of the cyano group in **48** to an amidine gave **49**.

### Structure‐Activity Relationship Studies

Guided by the docking studies, we set out to establish SAR by dissecting the main molecular features of the compound class. In addition to trypsin, thrombin, and factor Xa, we included KLK4, KLK7, and KLK8 as additional targets for selectivity analysis (Table 3). Having already determined the active stereoisomer **5**, we assessed the importance of the oxygen atoms in the oxazolidinone ring by testing lactam **43** and pyrrolidine **47**. While **43** showed similar potency and selectivity toward KLK6 relative to **5**, **47** slightly lost potency and selectivity against KLK6, indicating some binding role for the carbonyl. A much larger effect was observed with **49**, which lacks the amide carbonyl and results in a 40‐fold KLK6 potency loss. This data is largely consistent with the docking model, where the amide makes H‐bonds, while the oxazolidinone heteroatoms make no strong interactions with surrounding residues.

**Table 3:** Activity of synthesized inhibitors against KLK6 and related proteases.

Cmpd	Structure	**KLK6** ^a^	KLK4^a^	KLK7^a^	KLK8^a^	Trypsin^a^	Thrombin^a^	Factor Xa^a^
**5**	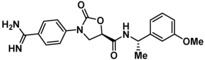	**6.6**	5.1	<4.7	5.0	5.3	<4.7	<4.7
**11**	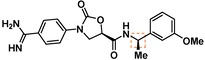	**4.8**	–	<4.7	<4.7	<4.7	<4.7	<4.7
**43**	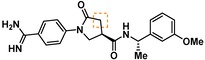	**6.5**	5.1	<4.7	<4.7	5.3	<4.7	<4.7
**47**	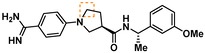	**6.1**	5.9	<4.7	5.1	5.3	<4.7	<4.7
**49**	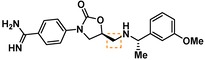	**5.0**	–	<4.7	<4.7	<4.7	<4.7	<4.7
**23**	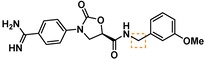	**5.4**	–	<4.7	<4.7	<4.7	<4.7	<4.7
**24**	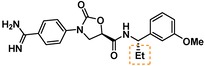	**7.5**	5.6	<4.7	5.3	5.8	<4.7	<4.7
**25**	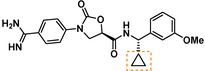	**7.5**	–	<4.7	6.1	6.6	<4.7	<4.7
**26**	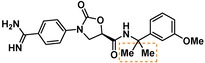	**6.8**	–	–	<4.7	5.0	–	–
**27**	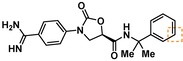	**6.1**	4.4	<4.7	<4.7	<4.7	<4.7	<4.7
**28**	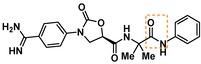	**7.5**	5.8	–	–	5.2	–	–
**29**	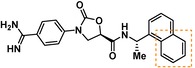	**7.9**	6.2	<4.7	6.0	6.1	<4.7	<4.7
**30**	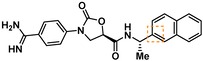	**7.0**	5.5	<4.7	5.0	5.4	<4.7	<4.7
**31**	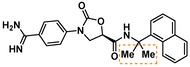	**7.1**	–	–	<4.7	5.3	–	–
**32**	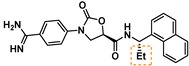	8.6*	6.8	<4.7	6.7	7.2	<4.7	<4.7
**33**	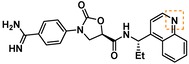	**6.9**	–	–	<4.7	5.2	–	–
**34**	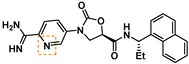	**8.3**	–	–	6.3	6.2	–	–
**35**	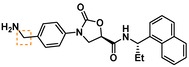	**6.5**	5.3	–	5.0	5.3	–	–

^a^ All values are reported as pIC_50_. *8.6, i. e. 2.5 nM, represents the assay limit in the KLK6 assay. SAR highlight of each compound is given by the dashed orange box. An entry of “−“ means the compound was not measured in that assay.

We next examined variations of the amide N‐substituents, which are hypothesized to bind in the S1′/S2′ pockets. As mentioned above, docking models suggested these were regions where subtle differences between KLK6 and the related proteases could be harnessed to boost selectivity. In particular, the small methyl group of **5** was predicted to fit into the S1′ pocket. This pocket is shallower in KLK6 than in related proteases, but our model suggested that it could accommodate a slightly larger group, strengthening this interaction. Consistent with the model, we found that removal of this methyl group (cmpd. **23**) is detrimental for activity, while replacement with an ethyl group (cmpd. **24**) results in an almost 10‐fold gain in potency for KLK6 and an increase in selectivity. A larger cyclopropane substituent (cmpd. **25**) does not improve the potency against KLK6 relative to **24**, and is deleterious for selectivity against trypsin and KLK8. It may be that the cyclopropyl group can better interact with the larger S1′ pocket of trypsin and KLK8. The appropriate stereochemistry is essential to direct the P1′ substituent towards the S1′ pocket, as testified by the loss of potency of diastereoisomer **11**. Interestingly, a dimethyl group, which presumably still directs one methyl group to the S1′ pocket, is also tolerated and appears to be beneficial for selectivity (cmpd. **26**).

The SAR of the P2′ substituent of the inhibitor class was investigated next. Compound **28** was synthesized to validate the docking prediction, which was indeed supported by an increase in potency compared to parent compound **27**, suggesting that the additional amide moiety might form further H‐bonds with the active site of KLK6. Moreover, **28** exhibited excellent selectivity over KLK4 and trypsin. In parallel, larger lipophilic substituents were introduced at P2′. Compound **29**, with a 1‐naphthyl P2′, was more potent than **28** (7.9 vs. 7.5 pIC_50_). Aiming to minimize the peptidic‐nature of the compounds, we continued building on **29**. Interestingly, the 1‐naphthyl regioisomer (**29**) was preferred over the 2‐naphthyl (**30**), which showed a 10‐fold potency loss. While a dimethyl P1′ substituent showed promise in the case of **26**, the gem‐dimethyl derivative **31** was ∼6 fold less potent than **29**. Fortunately, the optimal P1′ (ethyl) and P2′ (1‐naphthyl) substituents showed additive effects, with compound **32** approaching the potency limit of the KLK6 assay (pIC_50_=8.6). This compound retained a similar selectivity profile to the original hit **5**, while having almost 100‐fold higher potency against KLK6. Interestingly, introduction of a nitrogen atom in the naphthalene bicycle resulted in a dramatic loss of potency as measured for compound **33**, further highlighting the steep SAR observed within the P2′ substituent.

With a potent compound such as **32** in hand, we examined the possibility of lowering the basicity of the P1 amidino group, which could have an effect on permeability. Previous attempts to introduce less basic functionalities, e. g. benzylamine, amide and aminoisoquinoline within the **5** scaffold, resulted in significant to complete loss of activity against the target protease (data not shown). When the benzylamine replacement was made on the improved scaffold of **32**, it resulted in ∼100‐fold potency loss (cmpd **35**). However, good activity was still detected against KLK6 (pIC_50_=6.5) with an altogether unchanged selectivity profile, which provides a good starting point for further development of amidine‐free KLK6 inhibitors. Interestingly, introduction of a nitrogen atom in the benzamidine ring (cmpd **34**), which is predicted to reduce the pKa of the amidine from ∼11 to ∼9, resulted in a compound of slightly reduced potency for KLK6, but an improved selectivity profile against the related enzymes. An increase in selectivity over trypsin was also observed when the indole of **DKFZ**‐**251** was replaced with a 7‐azaindole.^21^ The structural reasons for these effects and whether they are connected is currently under further investigation.

**Figure 4:**
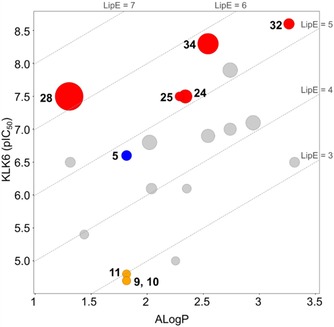
Lipophilicity plot of the compounds in this manuscript. The size of each dot represents selectivity over trypsin, with a larger dot indicating better selectivity. Trypsin data was used for this plot because trypsin data was generated for each compound and because trypsin activity varied significantly between derivatives. ALogP was calculated using Pipeline Pilot. Blue=hit substance. Yellow=stereoisomers of the hit. Red=potential leads.

When analyzing the compounds in a lipophilic efficiency (LipE) plot (LipE=pIC_50_ – logP), a number of observations could be made (Figure 4). The value of an S1′ ethyl vs. methyl is clear when comparing compounds **5** (LipE=4.79) and **24** (LipE=5.16). The two highly potent compounds **32** and **34** show improved LipE values of 5.34 and 5.76, respectively. Interestingly, **28** shows the highest LipE value of 6.20, due to its relatively low lipophilicity. While the additional amide in **28** might be expected to pose a problem for cell permeability, particularly in conjunction with an amidine moiety in the same molecule, further medicinal chemistry optimization could focus on mimicking the amide with heterocycles or other H‐bond donors/acceptors.

### Lead Compound Pharmacokinetic Properties

The most potent inhibitor, **32**, was tested in a number of computational and experimental ADME profiling assays (Table 4). The free base of **32** was calculated to have an ALogP of 3.26, and its conjugate acid a p*K*_a_ of 11.3. The trifluoroacetate salt of **32** showed excellent solubility in both kinetic and thermodynamic assay formats, presumably a result of the basic amidine. Clearance measurements with both mouse and human microsomes was relatively low. Clearance in mouse hepatocytes correlated well with the microsomal clearance (2.3 vs 2.4 mL/min/g), suggesting no significant contribution from phase II pathways. Measurement of cytochrome P450 (CYP) activity with five different isozymes showed no significant inhibition by **32**. Only the 3A4 isozyme was inhibited with an IC_50_ of 4.8 μM. The compound showed no instability in mouse plasma over 3 h, and was found to bind protein plasma to an extent of 89 %.

**Table 4:** ADME Profiling of compound **32** ⋅ **TFA**.

ALogP	3.26
pK_a_	11.3
Kinetic Solubility	>250 μM
Thermodynamic Solubility	2.3 mM
Mouse CLint	2.4 mL/min/g
Human CLint	3.1 mL/min/g
Mouse Hep Cl	2.3 mL/min/g
CYP Inhibition IC_50_ 1A2/2C9/2C19/2D6/3A4	>10/>10/7.9/>10/4.8 μM
Protein plasma (mouse) binding (fraction bound)	89 %
Mouse Plasma Stability (t_1/2_)	>180 min

### Compound 32 Reduces Invasion of HCT116 Cells

**Figure 5:**
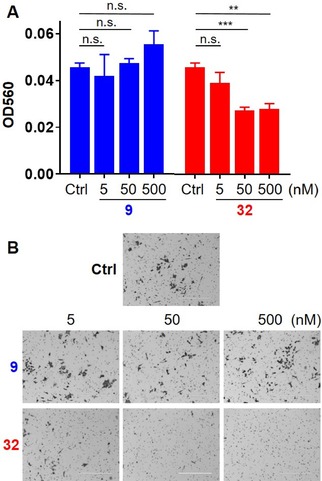
HCT116 cells were treated with **9** or **32** at the indicated concentrations and plated on Matrigel®‐coated filters. The upper chamber contained serum‐free medium, while the lower chamber contained 10 % fetal bovine serum. After 24 h, the number of invading cells was measured optically. (A) This figure is one representative example of four biological replicates, each with similar outcomes. Error bars are SEM. Technical replicates: DMSO and **9** at 5 nM (n=3); **9** at 50 and 500 nM and **32** at all concentrations (n=6). Statistical significance was calculated using an unpaired two‐tailed t‐test. n.s=p≥0.05; **=p<0.01; ***=p<0.001. (B) Representative images (Scale bar, 400 mm) of stained invaded cells outside of the inserts 24 h hours after treatment with **9**, **32**, or DMSO solvent control at the indicated concentrations. Phase contrast images were taken with EVOS FL Cell imagine system FL Auto microscope (Bio‐Rad).

We have previously shown that knockdown of KLK6 reduces migration and invasion of HCT116 cells in vitro.^13^ In order to test whether this effect could be recapitulated with small molecule KLK6 inhibitors, we measured the ability of **32** to reduce invasion of HCT116 cells. Because the enantiomer of **32** was never prepared, compound **9**, the enantiomer of hit **5**, was used as an inactive control substance. While compound **9** had no significant effect on the invasion of HCT116 cells after 24 h at multiple concentrations, compound **32** induced a significant reduction in invasion at both 50 nM and 500 nM (Figure 5). HCT116 cell growth and viability was not altered upon treatment with compounds **9** and **32** at the tested concentrations (data not shown).

## Conclusions

After a high throughput screen of the European Lead Factory compound collection, we discovered a validated hit cluster of *N*‐(4‐benzamidino)‐oxazolidinones that showed consistent inhibitory activity against KLK6. Docking‐guided optimization of this scaffold, with a focus on potency against KLK6 and selectivity against up to six different related proteases resulted in compounds with single digit nanomolar potency and good to excellent selectivity. Compound **32** was found to be the most potent inhibitor, while compounds **28** and **34** exhibited the highest selectivity against trypsin. ADME profiling of **32** showed that it has reasonable properties for pre‐clinical biological experiments. Finally, **32** was found to reduce invasion of HCT116 cells in a dose‐dependent manner, while **9**, a control substance from the inactive enantiomeric series, showed no such effect even at the highest tested concentrations.

These compounds show promise as useful chemical probes for the study of KLK6 biology. One particular benefit is the availability of inactive enantiomeric control compounds. While the enantiomers of **28**, **32**, and **34** were not synthesized as part of this ELF project, their synthesis is straightforward and will be made available upon request to interested scientists in the future.

## Experimental Section

**High Throughput Screen**: The European Lead Factory library of ∼350,000 compounds was used for high throughput screening at the Pivot Park Screening Center facilities in Oss, The Netherlands. HighRes Biosolutions robotic infrastructure using Cellario software was programmed to perform screening of 281 1536‐well plates. Using the primary KLK6 fluorescent intensity (FI) assay (see below), 7794 compounds having a Z‐score≤‐4 were selected for further follow‐up. An additional set of compounds was added based on Bayesian modeling to include potential false negatives in the hit confirmation. Of the tested 8706 compounds, 1026 showed >25 % inhibition when re‐tested in the KLK6 FI assay. When tested in serial dilutions (20 μM to 20 nM; 7‐points √10 diluted), 312 compounds showed a pEC_50_≥4.7. This set was subsequently tested in Trypsin, Thrombin and FXa assays (see below) resulting in a selection of 226 compounds showing KLK6 pEC_50_>6.0 OR 5.0<KLK6 pEC_50_<6.0 selectivity>0.1 OR 4.7<KLK6 pEC_50_<5.0 selectivity>1. Upon testing of these compounds using SPR and LC‐MS, a qualified hit list (QHL) was registered containing 50 compounds. For SPR, Biacore T200 was used and KLK6 was immobilized onto a CM5 chip. 226 test compounds were screened at 4 concentrations: 20, 4, 0.8 and 0.16 μM in running buffer: 50 mM Tris‐HCl, 1 mM EDTA pH 7.5, 150 mM NaCl, 0.05 % Tween 20, 3 % DMSO at 25 °C. Control injections of 5 μM **1** were used throughout the screen to ensure that KLK6 was stable and had not been blocked by potential irreversible binders/denaturants. An 8 point 1 in 3 dilution series of **1** (50 μM to 0.02 μM) was also applied at the beginning and end of each screening day.

**Docking Studies**: The Schrodinger Suite was used to prepare the KLK6 structure (PDB ID 4D8N) and to perform the docking experiments (Glide). Molecules were standardized and 3D conformers generated with the Ligprep module from the Schrodinger Suite using standard settings. Input stereochemistry was retained. The docking was performed using the Extra Precision (XP) method and the other parameters are assigned to their default values. A post‐docking refinement step was applied using the MM‐GBSA module that approximates the free energy of binding.

**Chemistry: (General)** Chemicals and solvents were from commonly used suppliers and were used without further purification. Silica gel 60 F254 analytical thin layer chromatography (TLC) plates were from Merck (Darmstadt, Germany) and visualized under UV light and/or with KMnO_4_ stain. Automated chromatography was performed with a Biotage Isolera Purification system (Uppsala, Sweden). Deuterated solvents were obtained from Cambridge Isotopes. All NMR spectra were recorded using a Bruker Avance 400 MHz spectrometer, and the residual solvent peak was used as internal reference (^1^H NMR: CHCl_3_ (7.26 ppm); DMSO (2.50 ppm); MeOH (3.31 ppm); ^13^C NMR: CHCl_3_ (77.16 ppm); DMSO (39.52 ppm); MeOH (49.00 ppm)). Chemical shifts (δ) are reported in ppm and coupling constants (*J*) in Hz. The following abbreviations were used for multiplicities: s=singlet, d=doublet, t=triplet, q=quartet, p=pentet, m=multiplet, br=broad. Preparative HPLC was carried out on a Waters HPLC comprising a Waters 2767 Sample Manager, Waters 2545 Binary Gradient Module, Waters Systems Fluidics Organiser, Waters 515 ACD pump, Waters 2998 Photodiode Array Detector, using a Waters XBridge Prep OBD C18, 5 μm, 19 mm×50 mm i.d. column and a flow rate of 20 mL/min. The general method used a gradient of 5 % acetonitrile/95 % water to 100 % acetonitrile (with 0.1 % trifluoroacetic acid in both phases). UV detection (254 nM) was used for the collection of fractions from HPLC. All final compounds were found to have ≥95 % purity, controlled by analytical (LC/MS) and confirmed by ^1^H NMR.

**Method A (amide coupling)**: To a mixture of carboxylic acid (1 equiv) and HATU (1.5 equiv) in DMF is added amine (1.2 equiv) and *i*‐Pr_2_NEt (3.0 equiv). After stirring for 24 h, the mixture is partitioned between EtOAc and brine/water. The layers are separated and the aqueous phase is extracted with EtOAc (2×). The combined organics are washed with brine (3×), dried (Na_2_SO_4_), filtered, concentrated under reduced pressure, and purified by chromatography.

**Method B (nitrile to amidine conversion)**: A mixture of benzonitrile (1 equiv), NH_2_OH ⋅ HCl (5 equiv), and *i*‐Pr_2_NEt (5 equiv) in EtOH is heated to 100 °C in a microwave for 60 min. After cooling, the mixture is diluted with MeOH and concentrated under reduced pressure. The residue is dissolved in AcOH and Ac_2_O (5 equiv), stirred at rt for 20 h, diluted with MeOH, and concentrated under reduced pressure. The residue is then dissolved in AcOH and Zn powder (10 equiv) is added with stirring for 2.5 h. The mixture is filtered, rinsing with MeOH, and the filtrate is concentrated, followed by azeotroping with heptane (x3). Purification by HPLC yields the product as a trifluoroacetate salt.

**Method C (sulfinyl imine formation)**: To a solution of (*R*)‐(+)‐2‐methylpropane‐2‐sulfinamide (**36**) (1 equiv) in CH_2_Cl_2_ is added pyridinium *p*‐toluenesulfonate (PPTS) (50 mol %), MgSO_4_ ⋅ H_2_O (5 equiv), and aldehyde (2 equiv). After 2 d, the resulting mixture is filtered, rinsed with CH_2_Cl_2_, and the filtrate is concentrated and purified by chromatography.

**Method D (Grignard addition to sulfinyl imine)**: To a solution of **37a** or **37c** (1 equiv) in CH_2_Cl_2_ is added a solution of EtMgBr (2 equiv) in Et_2_O at −78 °C under argon. After 4 h, the mixture is allowed to warm to rt. After 18 h, the reaction is quenched with H_2_O, diluted with EtOAc, and the two layers are separated. The aqueous layer is further extracted with EtOAc (2×), the combined organics are washed (brine), dried (MgSO_4_), filtered, concentrated, and purified by chromatography.

**(5*R*)‐3‐(4‐carbamimidoylphenyl)‐*N*‐[(*S*)‐1‐(3‐methoxyphenyl)ethyl]‐2‐oxooxazolidine‐5‐carboxamide (5)**: Method B with amide **21a** (78 mg, 0.21 mmol), NH_2_OH ⋅ HCl (71 mg, 1.0 mmol), *i*‐Pr_2_NEt (0.18 mL, 1.0 mmol) and EtOH (2.9 mL). Then AcOH (2.0 mL) and Ac_2_O (0.1 mL, 1.1 mmol). Then AcOH (2.0 mL) and Zn (0.14 g, 2.1 mmol). HPLC purification yielded 70 mg (66 %) of **5** **⋅** **TFA**: ^**1**^ **H NMR** (DMSO‐*d*_6_) δ: 9.25 (br s, 2H), 9.09 (br s, 2H), 8.97 (d, *J*=8.1 Hz, 1H), 7.85–7.91 (m, 2H), 7.77–7.83 (m, 2H), 7.24 (t, *J*=8.1 Hz, 1H), 6.88–6.93 (m, 2H), 6.81 (ddd, *J*=8.3, 2.5, 1.1 Hz, 1H), 5.17 (dd, *J*=9.3, 5.6 Hz, 1H), 4.89–5.00 (m, 1H), 4.36 (t, *J*=9.3 Hz, 1H), 4.08 (dd, *J*=9.3, 5.7 Hz, 1H), 3.73 (s, 3H), 1.40 (d, *J*=7.0 Hz, 3H) ppm. ^**13**^ **C NMR** (DMSO‐*d*_6_) δ: 166.8, 164.6, 159.3, 158.2 (q, *J*_*C‐F*_=30 Hz), 153.6, 145.5, 142.7, 129.4, 129.2, 122.3, 118.2, 117.4, 112.1, 111.8, 70.6, 55.0, 48.3, 47.5, 22.1 ppm. **HRMS** (ESI) *m/z*: (M+H)^+^ calcd for C_20_H_23_N_4_O_4_^+^: 383.1714; found: 383.1712.

**(5*R*)‐3‐(4‐carbamimidoylphenyl)‐*N*‐[(*R*)‐1‐(3‐methoxyphenyl)ethyl]‐2‐oxooxazolidine‐5‐carboxamide (11)**: Method B with amide **21b** (81 mg, 0.22 mmol), NH_2_OH ⋅ HCl (74 mg, 1.1 mmol), *i*‐Pr_2_NEt (0.19 mL, 1.1 mmol) and EtOH (3.0 mL). Then AcOH (2.0 mL) and Ac_2_O (0.10 mL, 1.1 mmol). Then AcOH (2.0 mL) and Zn (0.14 g, 2.2 mmol). HPLC purification yielded 65 mg (59 %) of **11** **⋅** **TFA**: ^**1**^ **H NMR** (DMSO‐*d*_6_) d: 9.24 (m, 3H), 8.96 (d, *J*=5.8 Hz, 1H), 7.93–7.84 (m, 2H), 7.83–7.76 (m, 2H), 7.24 (t, *J*=8.1 Hz, 1H), 6.95–6.89 (m, 2H), 6.81 (ddd, *J*=8.3, 2.5, 1.1 Hz, 1H), 5.21–5.12 (m, 1H), 4.95 (app p, *J*=7.1 Hz, 1H), 4.35 (t, *J*=9.6 Hz, 1H), 4.03 (dd, *J*=9.3, 5.8 Hz, 1H), 3.74 (s, 3H), 1.41 (d, *J*=7.0 Hz, 3H) ppm. ^**13**^ **C NMR** (DMSO‐*d*_6_) δ: 166.8, 164.7, 159.3, 153.6, 145.5, 142.7, 129.4, 129.2, 122.3, 118.3, 117.4, 112.1, 112.0, 70.5, 55.0, 48.3, 47.5, 22.2 ppm. **HRMS** (ESI) m/z: (M+H)^+^ calcd for C_20_H_23_N_4_O_4_^+^: 383.1714; found: 383.1713.

**Benzyl** ***N*** **‐(4‐cyanophenyl)carbamate (14)**: To a solution of 4‐aminobenzonitrile (**12**) (1.0 g, 8.5 mmol) in THF (17 mL) was added a solution of K_2_CO_3_ (2.3 g, 17 mmol) in water (17 mL), followed by slow addition of benzyl chloroformate (1.4 mL, 10 mmol). The resulting mixture was stirred at rt for 2.5 d, then partitioned between EtOAc and water. The two layers were separated and the organic layer was further washed with brine, dried (Na_2_SO_4_), filtered and concentrated under reduced pressure. The product was purified by flash chromatography (Telos 25 g, CH_2_Cl_2_ 100 %) to provide 2.0 g (95 %) of **14**: ^**1**^ **H NMR** (400 MHz, CDCl_3_), δ: 7.58–7.65 (m, 2H), 7.49–7.57 (m, 2H), 7.35–7.47 (m, 5H), 6.91 (br s, 1H), 5.24 (s, 2H) ppm.

**Benzyl (6‐cyanopyridin‐3‐yl)carbamate (15)**: To a suspension of 5‐aminopyridine‐2‐carbonitrile (**13**) (1.0 g, 8.4 mmol) and K_2_CO_3_ (1.7 g, 13 mmol) in THF (69 mL) was added benzyl chloroformate (1.8 mL, 13 mmol). The resulting mixture was stirred at rt for 2.5 days, then filtered rinsing with EtOAc. The filtrate was concentrated under reduced pressure, then purified by flash chromatography (ZIP 30 g, EtOAc in CH_2_Cl_2_, 0 to 10 %) to give 1.4 g (66 %) of **15**: ^**1**^ **H NMR** (CDCl_3_), δ: 8.55 (d, *J*=2.5 Hz, 1H), 8.21 (dd, *J*=8.4, 2.1 Hz, 1H), 7.68 (d, *J*=8.4 Hz, 1H), 7.33–7.49 (m, 5H), 7.02 (br s, 1H), 5.27 (s, 2H) ppm.

**4‐[(5*R*)‐5‐(hydroxymethyl)‐2‐oxo‐oxazolidin‐3‐yl]benzonitrile (17)**: To a cooled (−78 °C) solution of carbamate **14** (1.1 g, 4.4 mmol) in dry THF (44 mL) a solution of *n*‐BuLi (2.1 mL, 2.5 M in hexanes, 5.25 mmol) was added dropwise. After stirring the reaction mixture at −78 °C for 45 min, ((2*R*)‐oxiran‐2‐yl)methyl butanoate (**16**) (0.62 mL, 4.4 mmol) was added dropwise. The resulting mixture was stirred at −78 °C for 2 h and then allowed to warm to rt and stirred overnight. The reaction was quenched with saturated aqueous NH_4_Cl and extracted with EtOAc twice. The combined organic extracts were washed with brine, dried (Na_2_SO_4_), filtered and concentrated under reduced pressure. Chromatography (Telos 20 g, EtOAc in CH_2_Cl_2_, 0 to 75 %) gave 0.72 g (74 %) of **17**: ^**1**^ **H NMR** (CD_3_OD) δ: 7.76–7.85 (m, 2H), 7.69–7.76 (m, 2H), 4.73–4.81 (m, 1H), 4.17 (t, *J*=9.2 Hz, 1H), 3.97 (dd, *J*=9.0, 6.3 Hz, 1H), 3.87 (dd, *J*=12.6, 3.3 Hz, 1H), 3.70 (dd, *J*=12.6, 3.3 Hz, 1H) ppm.

**(5*R*)‐5‐(5‐(hydroxymethyl)‐2‐oxooxazolidin‐3‐yl)picolinonitrile (18)**: To a cooled (−78 °C) solution of carbamate **15** (0.30 g, 1.2 mmol) in dry THF (12 mL) was added a solution of *n*‐BuLi (0.57 mL, 2.5 M in hexanes, 1.43 mmol) dropwise. After stirring at −78 °C for 45 min, ((2*R*)‐oxiran‐2‐yl)methyl butanoate (**16**) (0.17 mL, 1.2 mmol) was added dropwise. The resulting mixture was stirred at −78 °C for 2 h and then allowed to warm to rt and stirred overnight. The reaction was quenched with saturated aqueous NH_4_Cl and extracted with EtOAc (x2). The combined organic extracts were washed with brine, dried (Na_2_SO_4_), filtered and concentrated under reduced pressure. Chromatography (ZIP‐30 g, EtOAc in heptane, 0–100 %, then MeOH in CH_2_Cl_2_) gave a material that was triturated with EtOH and a white solid collected by filtration, further rinsed with cold EtOH then dried on the filter pad to give 100 mg (39 %) of **18**: ^**1**^ **H NMR** (DMSO‐*d*6) δ: 8.96 (d, *J*=2.5 Hz, 1H), 8.21 (dd, *J*=8.7, 2.6 Hz, 1H), 8.06 (d, *J*=8.7 Hz, 1H), 5.25 (t, *J*=5.4 Hz, 1H), 4.81 (qd, *J*=6.2, 3.5 Hz, 1H), 4.14–4.22 (m, 1H), 3.93 (dd, *J*=9.0, 6.0 Hz, 1H), 3.65–3.75 (m, 1H), 3.53–3.64 (m, 1H) ppm. **LCMS** *m/z*=220.0 (M+H)^+^.

**(5*R*)‐3‐(4‐cyanophenyl)‐2‐oxo‐oxazolidine‐5‐carboxylic acid (19)**: To a cooled (0 °C) solution of alcohol **17** (0.72 g, 3.3 mmol) in MeCN/water (1 : 1, 18 mL) was added iodobenzene diacetate (2.7 g, 8.2 mmol) followed by TEMPO (0.10 g, 0.66 mmol). The resulting mixture was stirred at rt for 18 h, then partitioned between a saturated aqueous solution of NaHCO_3_ and EtOAc. The layers were separated and the organic layer was further washed with a saturated aqueous solution of NaHCO_3_. The aqueous extract was acidified with 5 M HCl and extracted with EtOAc (x3). The combined acidic organic extracts were dried (Na_2_SO_4_), filtered and concentrated under reduced pressure to give 730 mg (96 %) of **19**: ^**1**^ **H NMR** (DMSO‐*d*6), δ: 13.78 (br s, 1H), 7.87 (d, *J*=8.8 Hz, 2H), 7.77 (d, *J*=8.8 Hz, 2H), 5.24 (dd, *J*=9.8, 5.3 Hz, 1H), 4.40 (dd, *J*=9.8, 9.4 Hz, 1H), 4.18 (dd, *J*=9.4, 5.3 Hz, 1H) ppm.

**(5*R*)‐3‐(6‐cyanopyridin‐3‐yl)‐2‐oxooxazolidine‐5‐carboxylic acid (20)**: To a cooled (0 °C) solution of alcohol **18** (99 mg, 0.45 mmol) in MeCN/water (1 : 1, 2.4 mL) was added iodobenzene diacetate (0.36 g, 1.1 mmol) followed by TEMPO (14 mg, 90 μmol). The resulting mixture was stirred at rt for 48 h then partitioned between a saturated aqueous solution of. NaHCO_3_ and EtOAc. The organic extracts were further washed with a saturated aqueous solution of NaHCO_3_. The aqueous extract was acidified with 5 M HCl and extracted with EtOAc (x3). The combined acidic organic extracts were dried (Na_2_SO_4_), filtered and concentrated under reduced pressure to give 100 mg (97 %) of **20**: ^**1**^ **H NMR** (CD_3_OD) δ: 8.91–9.00 (m, 1H), 8.14–8.27 (m, 1H), 7.82–7.93 (m, 1H), 5.25 (dd, *J*=9.8, 5.3 Hz, 1H), 4.41–4.53 (m, 1H), 4.22–4.33 (m, 1H) ppm.

**(5*R*)‐3‐(4‐cyanophenyl)‐*N*‐[(1*S*)‐1‐(3‐methoxyphenyl)ethyl]‐2‐oxo‐oxazolidine‐5‐carboxamide (21a)**: Method A with **19** (75 mg, 0.32 mmol), HATU (0.18 g, 0.48 mmol), DMF (2.0 mL), (*S*)‐1‐(3‐methoxyphenyl)ethan‐1‐amine (**42**) (60 μL, 0.39 mmol), and *i*‐Pr_2_NEt (0.17 mL, 0.97 mmol). Chromatography: (ZIP‐10 g, EtOAc in heptane, 0–60 % then ZIP‐10 g, EtOAc in CH_2_Cl_2_, 0–10 %) gave 92 mg (78 %) of **21a**: ^**1**^ **H NMR** (CD_3_OD) δ: 7.69–7.82 (m, 4H), 7.21 (t, *J*=7.9 Hz, 1H), 6.85–6.94 (m, 2H), 6.78 (dd, *J*=8.3, 1.8 Hz, 1H), 5.14 (dd, *J*=9.5, 5.8 Hz, 1H), 5.05 (p, *J*=7.0 Hz, 1H), 4.35 (t, *J*=9.3 Hz, 1 H), 4.14 (dd, *J*=9.2, 5.9 Hz, 1H), 3.75 (s, 3H), 1.50 (d, *J*=7.0 Hz, 3H) ppm.

**(5*R*)‐3‐(4‐cyanophenyl)‐*N*‐[(1*R*)‐1‐(3‐methoxyphenyl)ethyl]‐2‐oxooxazolidine‐5‐carboxamide (21b)**: Method A with **19** (75 mg, 0.32 mmol), (*R*)‐1‐(3‐methoxyphenyl)ethan‐1‐amine (59 mg, 0.39 mmol), HATU (0.18 g, 0.48 mmol), DMF (2.5 mL), and *i*‐Pr_2_NEt (0.17 mL, 0.97 mmol). Chromatography (ZIP‐10 g, EtOAc in heptane, 0–60 %; then ZIP‐10 g, EtOAc in CH_2_Cl_2_, 0–10 %) gave 96 mg (81 %) of **21b**: ^**1**^ **H NMR** (400 MHz,CD_3_OD) δ: 7.69–7.83 (m, 4H) 7.24 (t, *J*=8.0 Hz, 1H) 6.89–6.97 (m, 2H) 6.75–6.86 (m, 1H) 5.11 (dd, *J*=9.5, 6.0 Hz, 1H) 5.05 (q, *J*=7.0 Hz, 1H) 4.35 (t, *J*=9.4 Hz, 1H) 4.12 (dd, *J*=9.3, 6.0 Hz, 1H) 3.79 (s, 3H) 1.50 (d, *J*=7.0 Hz, 3H) ppm.

**(5*R*)‐3‐(4‐cyanophenyl)‐*N*‐(3‐methoxybenzyl)‐2‐oxooxazolidine‐5‐carboxamide (21c)**: Method A with **19** (69 mg, 0.30 mmol), (3‐methoxyphenyl)methanamine (50 μL, 0.36 mmol), HATU (0.17 g, 0.45 mmol), DMF (1.5 mL), and *i‐*Pr_2_NEt (0.16 mL, 0.89 mmol). Chromatography (ZIP‐10 g, EtOAc in heptane, 0–100 %) gave 64 mg (60 %) of **21c**: ^**1**^ **H NMR** (DMSO‐*d*6) δ: 9.00 (t, *J*=5.9 Hz, 1H), 7.83–7.91 (m, 2H), 7.74–7.83 (m, 2H), 7.18–7.29 (m, 1H), 6.75–6.90 (m, 3H), 5.18 (dd, *J*=9.4, 5.6 Hz, 1H), 4.36 (t, *J*=9.3 Hz, 1H), 4.31 (d, *J*=6.0 Hz, 2H), 4.10 (dd, *J*=9.3, 5.8 Hz, 1H), 3.72 (s, 3H) ppm.

**(5*R*)‐3‐(4‐cyanophenyl)‐*N*‐[(1*S*)‐1‐(3‐methoxyphenyl)propyl]‐2‐oxo‐oxazolidine‐5‐carboxamide (21d)**: Method A with **19** (50 mg, 0.22 mmol), **39a** **⋅** **HCl** (52 mg, 0.26 mmol), HATU (0.12 g, 0.32 mmol), DMF (1.3 mL), and *i*‐Pr_2_NEt (0.15 mL, 0.86 mmol). Chromatography (ZIP‐10 g, EtOAc in heptane, 0–75 %) gave 75 mg (94 %) of **21d**: ^**1**^ **H NMR** (CD_3_OD) δ: 7.69–7.81 (m, 4H), 7.15–7.27 (m, 1H), 6.84–6.92 (m, 2H), 6.73–6.82 (m, 1H), 5.16 (dd, *J=*9.4, 5.9 Hz, 1H), 4.79 (t, *J=*7.5 Hz, 1H), 4.35 (t, *J=*9.3 Hz, 1H), 4.05–4.15 (m, 2H), 3.75 (s, 3H), 1.79–1.92 (m, 2H), 1.24 (t, *J=*7.1 Hz, 1H), 0.93 (t, *J=*7.4 Hz, 3H) ppm.

**(5*R*)‐3‐(4‐cyanophenyl)‐*N*‐((*S*)‐cyclopropyl(3‐methoxyphenyl)methyl)‐2‐oxooxazolidine‐5‐carboxamide (21e)**: Method A with **19** (0.10 g, 0.43 mmol), (±)‐cyclopropyl(3‐methoxyphenyl)methanamine ⋅ HCl (110 mg, 0.52 mmol), HATU (0.25 g, 0.65 mmol), DMF (4.0 mL), and *i*‐Pr_2_NEt (0.30 mL, 1.7 mmol). Chromatography twice (Telos‐20 g, EtOAc in heptane, 0–50 %) gave54 mg (32 %) of the desired diastereoisomer **21e** (second eluting spot). The stereochemistry of **21e** was assigned by advancing both diastereomers to final products and determining which was the active inhibitor: ^**1**^ **H NMR** (DMSO‐*d*_6_) δ: 9.09 (d, *J*=8.3 Hz, 1H), 7.82–7.92 (m, 2H), 7.72–7.82 (m, 2H), 7.17–7.30 (m, 1H), 6.90–6.99 (m, 2H), 6.76–6.87 (m, 1H), 5.17 (dd, *J*=9.5, 5.8 Hz, 1H), 4.31–4.42 (m, 1H), 4.19 (t, *J*=8.8 Hz, 1H), 4.02 (dd, *J*=9.3, 5.8 Hz, 1H), 3.68–3.77 (m, 3H), 1.13–1.31 (m, 2H), 0.45–0.58 (m, 2H), 0.27–0.43 (m, 2H) ppm.

**(5*R*)‐3‐(4‐cyanophenyl)‐*N*‐(2‐(3‐methoxyphenyl)propan‐2‐yl)‐2‐oxooxazolidine‐5‐carboxamide (21f)**: Method A with **19** (0.14 g, 0.60 mmol), 2‐(3‐methoxyphenyl)propan‐2‐amine^27^ (0.12 g, 0.72 mmol), HATU (0.34 g, 0.90 mmol), DMF (3.6 mL) and *i*‐Pr_2_NEt (0.42 mL, 2.4 mmol). Chromatography twice (ZIP‐10 g, EtOAc in heptane, 0–75 %) gave 100 mg (44 %) of **21f**: ^**1**^ **H NMR** (CD_3_OD) δ: 7.69–7.83 (m, 4H), 7.20 (t, *J*=8.0 Hz, 1H), 6.87–7.00 (m, 2H), 6.76 (dd, *J*=8.0, 2.1 Hz, 1H), 5.10 (dd, *J*=9.3, 5.8 Hz, 1H), 4.32 (t, *J*=9.3 Hz, 1H), 4.03–4.13 (m, 1H), 3.74 (s, 3H), 1.69 (s, 3H), 1.67 (s, 3H) ppm.

**(5*R*)‐3‐(4‐cyanophenyl)‐2‐oxo‐*N*‐(2‐phenylpropan‐2‐yl)oxazolidine‐5‐carboxamide (21g)**: Method A with **19** (50 mg, 0.22 mmol), 2‐phenylpropan‐2‐amine (40 μL, 0.26 mmol), HATU (0.12 g, 0.32 mmol), DMF (1.3 mL), and *i*‐Pr_2_NEt (0.11 mL, 0.65 mmol). Chromatography (ZIP‐10 g, EtOAc in heptane, 0–60 %) gave 58 mg (78 %) of **21g**: ^**1**^ **H NMR** (DMSO*‐d*_6_) δ: 8.63 (s, 1H), 7.82–7.92 (m, 2H), 7.70–7.82 (m, 2H), 7.24–7.39 (m, 4H), 7.14–7.22 (m, 1H), 5.13 (dd, *J*=9.2, 5.6 Hz, 1H), 4.25–4.37 (m, 1H), 3.96 (dd, *J*=9.2, 5.6 Hz, 1H), 1.61 (s, 3H), 1.60 (s, 3H) ppm.

**(5*R*)‐3‐(4‐cyanophenyl)‐*N*‐(2‐methyl‐1‐oxo‐1‐(phenylamino)propan‐2‐yl)‐2‐oxooxazolidine‐5‐carboxamide (21h)**: Method A with **19** (70 mg, 0.30 mmol), 2‐amino‐2‐methyl‐*N*‐phenyl‐propanamide (64 mg, 0.36 mmol), HATU (0.14 g, 0.36 mmol), DMF (2.8 mL), and *i*‐Pr_2_Net (0.21 mL, 1.2 mmol). Chromatography (ZIP‐10 g, EtOAc in heptane, 0–100 %) gave 0.11 g (93 %) of **21h**: ^**1**^ **H NMR** (CD_3_OD) δ: 7.73–7.79 (m, 2H), 7.67–7.73 (m, 2H), 7.43–7.50 (m, 2H), 7.22–7.32 (m, 2H), 7.04–7.12 (m, 1H), 5.15 (dd, *J*=9.5, 6.0 Hz, 1H), 4.34 (t, *J*=9.4 Hz, 1H), 4.11–4.16 (m, 1H), 1.62 (s, 3H), 1.61 (s, 3H) ppm.

**(5*R*)‐3‐(4‐cyanophenyl)‐*N*‐((*S*)‐1‐(naphthalen‐1‐yl)ethyl)‐2‐oxooxazolidine‐5‐carboxamide (21i)**: Method A with **19** (50 mg, 0.22 mmol), (1 *S*)‐1‐(1‐naphthyl)ethanamine (40 μL, 0.26 mmol), HATU (0.12 g, 0.32 mmol), DMF (1.3 mL), and *i*‐Pr_2_NEt (0.11 mL, 0.65 mmol). Chromatography (ZIP‐10 g, EtOAc in heptane, 0–100 %) gave 79 mg, (95 %) of **21i**: ^**1**^ **H NMR** (DMSO‐*d*_6_) δ: 9.11 (d, *J*=8.0 Hz, 1H), 8.10 (d, *J*=8.3 Hz, 1H), 7.92–7.98 (m, 1H), 7.82–7.89 (m, 3H), 7.72–7.79 (m, 2H), 7.47–7.61 (m, 4H), 5.70–5.81 (m, 2H), 5.16 (dd, *J*=9.3, 5.5 Hz, 1H), 4.35 (t, *J*=9.3 Hz, 1H), 4.04–4.11 (m, 1H), 1.56 (d, *J*=7.0 Hz, 3H) ppm.

**(5*R*)‐3‐(4‐cyanophenyl)‐*N*‐((*S*)‐1‐(naphthalen‐2‐yl)ethyl)‐2‐oxooxazolidine‐5‐carboxamide (21j)**: General Method A with **19** (0.10 g, 0.43 mmol), (1*S*)‐1‐(2‐naphthyl)ethanamine (88 mg, 0.52 mmol), HATU (0.24 g, 0.65 mmol), DMF (1.5 mL), and *i*‐Pr_2_NEt (0.23 ml, 1.3 mmol). Chromatography (Telos 4 g, 0–100 % EtOAc in DCM) gave a solid which was triturated with MeOH, filtered, and rinsed with cold EtOH. The solid was further dried under vacuum at 50 °C to give 0.14 g (83 %) of **21j**: ^**1**^ **H NMR** (DMSO‐*d_6_*) δ: 9.04 (d, *J*=7.8 Hz, 1H), 7.81–7.93 (m, 6H), 7.75–7.81 (m, 2H), 7.44–7.57 (m, 3H), 5.08–5.21 (m, 2H), 4.34 (t, *J*=9.3 Hz, 1H), 4.03 (dd, *J*=9.3, 5.8 Hz, 1H), 1.52 (d, *J*=7.0 Hz, 3H) ppm.

**(5*R*)‐3‐(4‐cyanophenyl)‐*N*‐(2‐(naphthalen‐1‐yl)propan‐2‐yl)‐2‐oxooxazolidine‐5‐carboxamide (21k)**: Method A with **19** (70 mg, 0.30 mmol), 2‐(naphthalen‐1‐yl)propan‐2‐amine (67 mg, 0.36 mmol), HATU (0.17 g, 0.45 mmol), DMF (1.8 mL), and *i*‐Pr_2_NEt (0.21 mL, 1.2 mmol). Chromatography (ZIP‐10 g, EtOAc in heptane, 0–65 %) gave 0.10 g (87 %) of **21k**: ^**1**^ **H NMR** (CD_3_OD) δ: 8.35–8.46 (m, 1H), 7.79–7.88 (m, 1H), 7.57–7.79 (m, 6H), 7.27–7.48 (m, 3H), 5.01 (dd, *J*=9.5, 5.3 Hz, 1H), 4.18 (t, *J*=9.3 Hz, 1H), 3.71–3.80 (m, 1H), 1.96 (s, 3H), 1.93 (s, 3H) ppm.

**(5*R*)‐3‐(4‐cyanophenyl)‐*N*‐((*S*)‐1‐(naphthalen‐1‐yl)propyl)‐2‐oxooxazolidine‐5‐carboxamide (21l)**: Method A with **19** (70 mg, 0.30 mmol), **39b** **⋅** **HCl** (80 mg, 0.36 mmol), HATU (0.17 g, 0.45 mmol), DMF (1.3 mL), and *i*‐Pr_2_NEt (0.21 mL, 1.2 mmol). Chromatography (Telos‐20 g, EtOAc in heptane, 0–50 %) gave 92 mg (76 %) of **21l**: ^**1**^ **H NMR** (CD_3_OD) δ: 8.13 (d, *J*=8.5 Hz, 1H) 7.82–7.91 (m, 1H) 7.78 (d, *J*=8.3 Hz, 1H), 7.65–7.75 (m, 4H) 7.37–7.58 (m, 4H) 5.70 (dd, *J*=8.7, 5.9 Hz, 1H) 5.19 (dd, *J*=9.2, 5.8 Hz, 1H) 4.36 (t, *J*=9.2 Hz, 1H) 4.11 (dd, *J*=9.3, 6.0 Hz, 1H) 1.94–2.14 (m, 2H) 1.06 (t, *J*=7.4 Hz, 3H) ppm.

**(5*R*)‐3‐(4‐cyanophenyl)‐2‐oxo‐*N*‐((*S*)‐1‐(quinolin‐4‐yl)propyl)oxazolidine‐5‐carboxamide (21m)**: Method A with **19** (70 mg, 0.30 mmol), **39c** **⋅** **HCl** (80 mg, 0.36 mmol), HATU (172 mg, 0.45 mmol), DMF (1.3 mL), and *i*‐Pr_2_NEt (0.21 mL, 1.2 mmol). Chromatography (Telos‐10 g, EtOAc in heptane, 20–100 %) gave 77 mg (37 %) of **21m**: ^**1**^ **H NMR** (CD_3_OD) δ: 8.82 (d, *J*=4.5 Hz, 1H), 8.26 (dt, *J*=8.4, 0.7 Hz, 1H), 8.02–8.09 (m, 1H), 7.69–7.81 (m, 5H), 7.65 (ddd, *J*=8.4, 6.9, 1.2 Hz, 1H), 7.57 (d, *J*=4.5 Hz, 1H), 5.71 (dd, *J*=9.0, 5.5 Hz, 1H), 5.17 (dd, *J*=9.5, 5.8 Hz, 1H), 4.34 (t, *J*=9.4 Hz, 1H), 4.14 (dd, *J*=9.3, 5.8 Hz, 1H), 1.92–2.10 (m, 2H), 1.06 (t, *J*=7.4 Hz, 3H) ppm.

**(5*R*)‐3‐(6‐cyanopyridin‐3‐yl)‐*N*‐((*S*)‐1‐(naphthalen‐1‐yl)propyl)‐2‐oxooxazolidine‐5‐carboxamide (22)**: Method A with **20** (50 mg, 0.21 mmol), **39b** **⋅** **HCl** (60 μL, 0.26 mmol), HATU (0.12 g, 0.32 mmol), DMF (1.3 mL), and *i*‐Pr_2_NEt (0.15 mL, 0.86 mmol). Preparative HPLC gave 58 mg (67 %) of **22**: ^**1**^ **H NMR** (DMSO‐*d*6) δ: 9.07 (d, *J*=8.03 Hz, 1H), 8.95 (d, *J*=2.5 Hz, 1H), 8.11–8.27 (m, 2H), 8.07 (d, *J*=8.8 Hz, 1H), 7.95 (d, *J*=7.8 Hz, 1H), 7.84 (d, *J*=7.8 Hz, 1H), 7.45–7.63 (m, 4H), 5.51–5.63 (m, 1H), 5.26 (dd, *J*=9.3, 5.8 Hz, 1H), 4.33–4.47 (m, 1H), 4.06–4.18 (m, 1H), 1.81–2.00 (m, 2H), 0.98 (t, *J*=7.3 Hz, 3H) ppm.

**(5*R*)‐3‐(4‐carbamimidoylphenyl)‐*N*‐(3‐methoxybenzyl)‐2‐oxooxazolidine‐5‐carboxamide (23)**: Method B with **21c** (62 mg, 0.18 mmol), NH_2_OH ⋅ HCl (59 mg, 0.85 mmol), *i*‐Pr_2_NEt (0.15 mL, 0.85 mmol) and EtOH (2.9 mL). Then AcOH (2.0 mL) and Ac_2_O (83 μL, 0.88 mmol). Then AcOH (2.0 mL) and Zn (0.15 g, 1.8 mmol). HPLC purification yielded 32 mg (38 %) of **23** **⋅** **TFA**: ^**1**^ **H NMR** (DMSO‐*d*_6_) δ: 9.26 (s, 2H), 9.14 (s, 2H), 9.05 (d, *J*=6.9 Hz, 1H), 7.96–7.85 (m, 2H), 7.85–7.77 (m, 2H), 7.24 (t, *J*=8.1 Hz, 1H), 6.88–6.80 (m, 3H), 5.25–5.17 (m, 1H), 4.43–4.35 (m, 1H), 4.32 (d, *J*=6.0 Hz, 2H), 4.12 (dd, *J*=9.3, 5.7 Hz, 1H), 3.72 (s, 3H) ppm. ^**13**^ **C NMR** (DMSO‐*d*_6_) δ: 167.9, 164.7, 159.3, 158.0 (q, *J*_*C‐F*_=30 Hz), 153.5, 142.7, 140.2, 129.4, 129.2, 122.4, 119.5, 117.5, 117.2 (q, *J*_*C‐F*_=300 Hz), 113.0, 112.4, 70.6, 55.0, 47.6, 42.2 ppm. **HRMS** (ESI) m/z: (M+H)^+^ calcd for C_19_H_21_N_4_O_4_^+^: 369.1557; found: 369.1557.

**(5*R*)‐3‐(4‐carbamimidoylphenyl)‐*N*‐[(1*S*)‐1‐(3‐methoxyphenyl)propyl]‐2‐oxo‐oxazolidine‐5‐carboxamide (24)**: Method B with **21d** (70 mg, 0.18 mmol), NH_2_OH ⋅ HCl (61 mg, 0.89 mmol), *i*‐Pr_2_NEt (0.15 mL, 0.89 mmol) and EtOH (3.3 mL). Then AcOH (3.0 mL), and Ac_2_O (90 μL, 0.92 mmol). Then AcOH (3.0 mL) and Zn (0.24 g, 3.7 mmol). HPLC purification gave a solid which was triturated with EtOH to give 52 mg (56 %) of **21d** **⋅** **TFA**: ^**1**^ **H NMR** (DMSO‐*d*_6_) δ: 9.20 (s, 4H), 8.93 (d, *J*=8.4 Hz, 1H), 7.92–7.84 (m, 2H), 7.83–7.78 (m, 2H), 7.27–7.21 (m, 1H), 6.94–6.87 (m, 2H), 6.83–6.77 (m, 1H), 5.20 (dd, *J*=9.3, 5.7 Hz, 1H), 4.70 (td, *J*=8.3, 6.5 Hz, 1H), 4.37 (t, *J*=9.3 Hz, 1H), 4.05 (dd, *J*=9.3, 5.7 Hz, 1H), 3.73 (s, 3H), 1.82–1.66 (m, 2H), 0.85 (t, *J*=7.3 Hz, 3H) ppm. ^**13**^ **C NMR** (DMSO‐*d*_6_) δ: 167.1, 164.7, 159.3, 158.5 (q, *J*_*C‐F*_=31 Hz), 153.6, 144.5, 142.7, 129.3, 129.2, 122.3, 118.8, 117.4, 117.2 (q, *J*_*C‐F*_=300 Hz), 112.3, 112.1, 70.6, 55.0, 54.5, 47.6, 28.9, 11.0 ppm. **HRMS** (ESI) m/z: (M+H)^+^ calcd for C_21_H_25_N_4_O_4_^+^: 397.1870; found: 397.1870.

**(5*R*)‐3‐(4‐carbamimidoylphenyl)‐*N*‐((1*S*)‐cyclopropyl(3‐methoxyphenyl)methyl)‐2‐oxooxazolidine‐5‐carboxamide (25)**: Method B with **21e** (52 mg, 0.13 mmol), NH_2_OH ⋅ HCl (44 mg, 0.64 mmol), *i*‐Pr_2_NEt (0.11 mL, 0.64 mmol) and EtOH (2.5 mL). Then AcOH (2.0 mL) and Ac_2_O (60 μL, 0.66 mmol). Then AcOH (2.0 mL) and Zn (87 mg, 1.3 mmol). HPLC purification gave 35 mg (50 %) of **25** **⋅** **TFA**: ^**1**^ **H NMR** (DMSO‐*d*_6_) δ: 9.24 (s, 2H), 9.12 (d, *J*=8.3 Hz, 1H), 8.95 (s, 2H), 7.85–7.92 (m, 2H), 7.77–7.85 (m, 2H), 7.20–7.29 (m, 1H), 6.92–6.99 (m, 2H), 6.82 (ddd, *J*=8.3, 2.4, 1.1 Hz, 1H), 5.20 (dd, *J*=9.4, 5.6 Hz, 1H), 4.34–4.42 (m, 1H), 4.19 (t, *J*=8.7 Hz, 1H), 4.05 (dd, *J*=9.3, 5.8 Hz, 1H), 3.69–3.77 (m, 3H), 1.19–1.29 (m, 1H), 0.52 (td, *J*=7.5, 5.1 Hz, 2H), 0.26–0.43 (m, 2H) ppm. **LCMS** *m/z*=409.2 [M+H]^+^.

**(5*R*)‐3‐(4‐carbamimidoylphenyl)‐*N*‐(2‐(3‐methoxyphenyl)propan‐2‐yl)‐2‐oxooxazolidine‐5‐carboxamide (26)**: Method B with **21f** (0.10 g, 0.26 mmol), NH_2_OH ⋅ HCl (88 mg, 1.3 mmol), *i*‐Pr_2_NEt (0.22 mL, 1.3 mmol) and EtOH (4.8 mL). Then AcOH (4.0 mL) and Ac_2_O (0.12 mL, 1.3 mmol). Then AcOH (4.0 mL) and Zn (0.34 g, 5.3 mmol). HPLC purification gave a solid which was triturated with cold EtOH and cold MeOH to give 49 mg (37 %) of **26** **⋅** **TFA**: ^**1**^ **H NMR** (DMSO*‐d*_6_) δ: 9.09 (br s, 4H), 8.65 (s, 1H), 7.74–7.93 (m, 4H), 7.21 (t, *J*=8.0 Hz, 1H), 6.84–6.98 (m, 2H), 6.77 (dd, *J*=8.0, 2.0 Hz, 1H), 5.16 (dd, *J*=9.2, 5.4 Hz, 1H), 4.34 (t, *J*=9.3 Hz, 1H), 3.98 (dd, *J*=9.2, 5.6 Hz, 1H), 3.72 (s, 3H), 1.61 (s,3H), 1.58 (s, 3H) ppm. **LCMS** *m/z*=397.2 [M+H]^+^.

**(5*R*)‐3‐(4‐carbamimidoylphenyl)‐2‐oxo‐*N*‐(2‐phenylpropan‐2‐yl)oxazolidine‐5‐carboxamide (27)**: Method B with **21g** (58 mg, 0.17 mmol), NH_2_OH ⋅ HCl (56 mg, 0.80 mmol), *i*‐Pr_2_NEt (0.14 mL, 0.18 mmol) and EtOH (2.3 mL). Then AcOH (2.0 mL) and Ac_2_O (80 μL, 0.84 mmol). Then AcOH (2.0 mL) and Zn (0.11 g, 1.7 mmol). Preparative HPLC gave 61 mg (76 %) of **27** **⋅** **TFA**: ^**1**^ **H NMR** (DMSO*‐d*_6_) δ: 9.24 (br s, 2H), 8.94 (br s, 2H), 8.67 (s, 1H), 7.76–7.92 (m, 4H), 7.24–7.42 (m, 4H), 7.14–7.24 (m, 1H), 5.16 (dd, *J*=9.2, 5.6 Hz, 1H), 4.34 (t, *J*=9.2 Hz, 1H), 3.99 (dd, *J*=9.3, 5.8 Hz, 1H), 1.63 (s, 3H), 1.59 (s, 3H) ppm. **LCMS** *m/z*=367 [M+H]^+^.

**(5*R*)‐3‐(4‐carbamimidoylphenyl)‐*N*‐(2‐methyl‐1‐oxo‐1‐(phenylamino)propan‐2‐yl)‐2‐oxooxazolidine‐5‐carboxamide (28)**: Method B with **21h** (0.11 g, 0.28 mmol), NH_2_OH ⋅ HCl (93 mg, 1.3 mmol), *i*‐Pr_2_Net (0.23 mL, 1.3 mmol) and EtOH (2.0 mL). Then AcOH (2.0 mL), and Ac_2_O (0.13 mL, 1.4 mmol). Then AcOH (2.0 mL) and Zn (0.18 g, 2.8 mmol). Preparative HPLC gave 48 mg (33 %) of **28** **⋅** **TFA**: ^**1**^ **H NMR** (DMSO‐*d*_6_) δ: 9.50 (s, 1H), 9.26 (s, 2H), 9.18 (s, 2H), 8.58 (s, 1H), 7.93–7.86 (m, 2H), 7.80 (d, *J*=9.0 Hz, 2H), 7.60–7.49 (m, 2H), 7.31–7.21 (m, 2H), 7.06–7.00 (m, 1H), 5.26–5.13 (m, 1H), 4.38 (t, *J*=9.4 Hz, 1H), 4.09–3.98 (m, 1H), 1.50 (s, 3H), 1.49 (s, 3H) ppm. ^**13**^ **C NMR** (DMSO‐*d*_6_) δ: 172.1, 167.3, 164.7, 158.5 (q, *J*_*C‐F*_=33 Hz), 153.6, 142.7, 139.1, 129.3, 128.4, 123.3, 122.3, 120.3, 117.4, 116.9 (q, *J*_*C‐F*_=297 Hz), 70.3, 56.9, 47.6, 24.8, 24.6 ppm. **HRMS** (ESI) *m/z*: (M+H)^+^ calcd for C_21_H_24_N_5_O_4_^+^: 410.1823; found: 410.1823.

**(5*R*)‐3‐(4‐carbamimidoylphenyl)‐*N*‐((*S*)‐1‐(naphthalen‐1‐yl)ethyl)‐2‐oxooxazolidine‐5‐carboxamide (29)**: Method B with amide **21i** (78 mg, 0.20 mmol), NH_2_OH ⋅ HCl (67 mg, 0.97 mmol), *i*‐Pr_2_NEt (0.17 mL, 0.97 mmol) and EtOH (2.7 mL). Then AcOH (2.0 mL) and Ac_2_O (0.10 mL, 1.0 mmol). Then AcOH (2.0 mL) and Zn (0.13 g, 2.0 mmol). Preparative HPLC gave a solid which was triturated with cold EtOH to give 32 mg (30 %) of **29** **⋅** **TFA**: ^**1**^ **H NMR** (DMSO‐*d*_6_) δ: 9.24 (s, 2H), 9.15 (d, *J*=7.8 Hz, 1H), 8.89 (br s, 2H), 8.11 (d, *J*=8.0 Hz, 1H), 7.91–7.99 (m, 1H), 7.75–7.91 (m, 4H), 7.46–7.63 (m, 4H), 5.76 (p, *J*=7.0 Hz, 1H), 5.19 (dd, *J*=9.3, 5.8 Hz, 1H), 4.37 (t, *J*=9.2 Hz, 1H), 4.10 (dd, *J*=9.3, 5.8 Hz, 1H), 1.56 (d, *J*=7.0 Hz, 3H) ppm. **LCMS** *m/z*=403.0 [M+H]^+^.

**(5*R*)‐3‐(4‐carbamimidoylphenyl)‐*N*‐[(1*S*)‐1‐(2‐naphthyl)ethyl]‐2‐oxo‐oxazolidine‐5‐carboxamide (30)**: Method B with **21j** (60 mg, 0.16 mmol), NH_2_OH ⋅ HCl (52 mg, 0.75 mmol), *i*‐Pr_2_NEt (0.13 ml, 0.75 mmol) and EtOH (2.5 mL). Then AcOH (2.0 mL) and Ac_2_O (70 μl, 0.78 mmol). Then AcOH (2.0 mL) and Zn (0.10 mg, 1.6 mmol). Preparative HPLC gave 26 mg (41 %) of **30** **⋅** **TFA**: ^**1**^ **H NMR** (DMSO‐*d*_6_) δ: 9.24 (s, 2H), 9.08 (d, *J*=8.0 Hz, 1H), 8.90 (br s, 2H), 7.84–7.93 (m, 5H), 7.76–7.84 (m, 3H), 7.43–7.57 (m, 3H), 5.11–5.23 (m, 2H), 4.37 (t, *J*=9.4 Hz, 1H), 4.10 (dd, *J*=9.4, 5.6 Hz, 1H), 1.52 (d, *J*=7.0 Hz, 3H) ppm. **LCMS** *m/z*=403.2 [M+H]^+^.

**(5*R*)‐3‐(4‐carbamimidoylphenyl)‐*N*‐(2‐(naphthalen‐1‐yl)propan‐2‐yl)‐2‐oxooxazolidine‐5‐carboxamide (31)**: Method B with **21k** (0.10 g, 0.26 mmol), NH_2_OH ⋅ HCl (85 mg, 1.2 mmol), *i*‐Pr_2_NEt (0.21 mL, 1.2 mmol), and EtOH (4.6 mL). Then AcOH (3.9 mL) and Ac_2_O (0.12 mL, 1.3 mmol). Then AcOH (3.0 mL) and Zn (0.33 g, 5.1 mmol). Preparative HPLC gave a solid which was triturated with cold EtOH to give 62 mg (46 %) of **31** **⋅** **TFA**: ^**1**^ **H NMR** (DMSO*‐d*_6_) δ: 9.22 (s, 2H), 9.06 (s, 1H), 8.89 (br s, 2H), 8.47–8.57 (m, 1H), 7.87–7.96 (m, 1H), 7.74–7.87 (m, 3H), 7.69 (d, *J*=9.0 Hz, 2H), 7.56 (d, *J*=7.0 Hz, 1H), 7.39–7.50 (m, 3H), 5.10 (dd, *J*=9.3, 5.3 Hz, 1H), 4.26 (t, *J*=9.3 Hz, 1H), 3.68–3.80 (m, 1H), 1.85 (s, 3H), 1.84 (s, 3H) ppm. **LCMS** *m/z*=417.2. [M+H]^+^.

**(5*R*)‐3‐(4‐carbamimidoylphenyl)‐*N*‐((*S*)‐1‐(naphthalen‐1‐yl)propyl)‐2‐oxooxazolidine‐5‐carboxamide (32)**: Method B with **21l** (90 mg, 0.22 mmol), NH_2_OH ⋅ HCl (75 mg, 1.1 mmol), *i*‐Pr_2_NEt (0.19 mL, 1.1 mmol) and EtOH (2.5 mL). Then AcOH (2.0 mL) and Ac_2_O (0.11 mL, 1.1 mmol). Then AcOH (2.0 mL) and Zn (0.15 g, 2.2 mmol). Preparative HPLC gave 50 mg (42 %) of **32** **⋅** **TFA**: ^**1**^ **H NMR** (DMSO‐*d*_6_) δ: 9.12 (br m, 4H), 9.10 (d, *J*=8.3 Hz, 1H), 8.18–8.11 (m, 1H), 7.94 (dd, *J*=7.7, 2.0 Hz, 1H), 7.89–7.82 (m, 3H), 7.82–7.76 (m, 2H), 7.59–7.48 (m, 4H), 5.61–5.50 (m, 1H), 5.23 (dd, *J*=9.3, 5.6 Hz, 1H), 4.44–4.34 (m, 1H), 4.06 (dd, *J*=9.3, 5.6 Hz, 1H), 1.96–1.86 (m, 2H), 0.97 (t, *J*=7.3 Hz, 3H) ppm. ^**13**^ **C NMR** (DMSO‐*d*_6_) δ: 167.3, 164.6, 158.2 (q, *J*_*C‐F*_=32 Hz), 153.6, 142.7, 138.7, 133.4, 130.5, 129.3, 128.8, 127.5, 126.3, 125.7, 125.5, 123.0, 122.9, 122.3, 117.4, 117.2 (q, *J*_*C‐F*_=300 Hz), 70.6, 50.4, 47.7, 28.5, 11.3 ppm. **HRMS** (ESI) *m/z*: (M+H)^+^ calcd for C_24_H_25_N_4_O_3_^+^: 417.1921; found: 417.1921.

**(5*R*)‐3‐(4‐carbamimidoylphenyl)‐2‐oxo‐*N*‐((1*S*)‐1‐(quinolin‐4‐yl)propyl)oxazolidine‐5‐carboxamide (33)**: A mixture of **21m** (0.14 g, 0.34 mmol), NH_2_OH ⋅ HCl (0.11 g, 1.6 mmol) and *i*‐Pr_2_NEt (0.28 mL, 1.6 mmol) in EtOH (2.0 mL) was heated to 100 °C in a microwave reactor for 60 min. After cooling, the reaction mixture was concentrated and purified by preparative HPLC to give 90 mg (71 %) of the corresponding amidoxime ((5*R*)‐3‐(4‐(*N*‐hydroxycarbamimidoyl)phenyl)‐2‐oxo‐*N*‐((*S*)‐1‐(quinolin‐4‐yl)propyl)oxazolidine‐5‐carboxamide) as a TFA salt: **LCMS** *m/z*=434.2 [M+H]^+^. This material (40 mg, 60 μmol) was mixed with AcOH (1.0 mL) and Ac_2_O (30 μL, 0.31 mmol). After 18 h, the reaction mixture was concentrated, azeotroping with heptane. The residue was redissolved in a mixture of MeOH/EtOAc (8.0 mL, 1 : 1) and subjected to hydrogenation using an H‐cube apparatus (Pd/C, rt, 10 bar, 1 mL/min). After concentration, HPLC purification gave 14 mg (29 %) of **33** **⋅** **TFA**: ^**1**^ **H NMR** (DMSO‐*d*_6_) δ: 9.20–9.34 (m, 3H), 8.98 (d, *J*=4.8 Hz, 3H), 8.34 (d, *J*=8.0 Hz, 1H), 8.11 (dd, *J*=8.5, 1.0 Hz, 1H), 7.83–7.90 (m, 3H), 7.76–7.83 (m, 2H), 7.73 (ddd, *J*=8.4, 6.9, 1.2 Hz, 1H), 7.66 (d, *J*=4.8 Hz, 1H), 5.57–5.66 (m, 1H), 5.25 (dd, *J*=9.4, 5.6 Hz, 1H), 4.37 (t, *J*=9.3 Hz, 1H), 4.03 (dd, *J*=9.4, 5.6 Hz, 1H), 1.85–1.96 (m, 2H), 0.94–1.04 (m, 3H) ppm. **LCMS** *m/z*=418.2 [M+H]^+^.

**(5*R*)‐3‐(6‐carbamimidoylpyridin‐3‐yl)‐*N*‐((1*S*)‐1‐(naphthalen‐1‐yl)propyl)‐2‐oxooxazolidine‐5‐carboxamide (34)**: A mixture of **22** (53 mg, 0.13 mmol), NH_2_OH ⋅ HCl (44.15 mg, 0.64 mmol) and *i*‐Pr_2_NEt (0.11 ml, 0.64 mmol) in EtOH (2.4 mL) was heated with microwave irradiation at 100 °C for 60 min. The mixture was concentrated and taken up in AcOH (2 mL) and Ac_2_O (0.06 ml, 0.66 mmol). After 18 h at rt, additional Ac_2_O (0.06 ml, 0.66 mmol) was added. After another 24 h, the acetylation reaction was not complete and the mixture was concentrated under reduced pressure, azeotroping with heptane (x3). Remaining “starting material” amidoxime was separated from the acetylated amidoxime product via preparative HPLC. The acetylated fractions were concentrated, dissolved in 1 : 1 EtOH/EtOAc (10 mL) and subjected to hydrogenation using an H‐cube apparatus (Pd/C, rt, 10 bar, 1 mL/min). After concentration, preparative HPLC gave **34** **⋅** **TFA**: ^**1**^ **H NMR** (DMSO‐*d*_6_) δ: 9.44 (br s, 2H), 9.19 (br s, 4H), 8.21–8.35 (m, 2H), 8.16 (d, *J*=8.0 Hz, 1H), 7.95 (d, *J*=7.5 Hz, 1H), 7.85 (d, *J*=8.0 Hz, 1H), 7.41–7.63 (m, 4H), 5.51–5.63 (m, 1H), 5.29 (dd, *J*=9.2, 5.9 Hz, 1H), 4.44 (t, *J*=9.4 Hz, 1H), 4.16 (dd, *J*=9.2, 5.9 Hz, 1H), 1.84–1.99 (m, 2H), 0.98 (t, *J*=7.3 Hz, 3H) ppm. **LCMS** *m/z*=418.2 [M+H]^+^.

**(5*R*)‐3‐(4‐(aminomethyl)phenyl)‐*N*‐[(1*S*)‐1‐(naphthalen‐1‐yl)propyl]‐2‐oxooxazolidine‐5‐carboxamide (35)**: A solution of amide **21l** (50 mg, 0.13 mmol) in MeOH/dioxane (1 : 1, 6.5 mL) was subjected twice to hydrogenation using H‐cube (50 bar) at 55 °C with a Raney‐Nickel column and a flow of 1 mL/min. The eluting solution was concentrated under reduced pressure, and the residue was purified by preparative HPLC to yield 20 mg (31 %) of **35** **⋅** **TFA**: ^**1**^ **H NMR** (DMSO‐*d*6) δ: 9.08 (d, *J*=8.3 Hz, 1H), 8.20 (br s, 3H), 8.16 (d, *J*=8.3 Hz, 1H), 7.95 (dd, *J*=8.2, 1.4 Hz, 1H), 7.84 (d, *J*=7.8 Hz, 1H), 7.58–7.65 (m, 2H), 7.41–7.58 (m, 5H), 5.51–5.61 (m, 1H), 5.19 (dd, *J*=9.4, 5.6 Hz, 1H), 4.33 (t, *J*=9.2 Hz, 1H), 3.96–4.05 (m, 3H), 1.86–1.98 (m, 2H), 0.98 (t, *J*=7.3 Hz, 3H) ppm. **LCMS** *m/z*=388.4 [M+H^+^−NH_3_]^+^.

**(*R*)‐*N*‐(3‐methoxybenzylidene)‐2‐methylpropane‐2‐sulfinamide (37a)**: Method C with **36** (0.50 g, 4.1 mmol), PPTS (52 mg, 0.21 mmol), MgSO_4_ ⋅ H_2_O (2.5 g, 21 mmol), 3‐methoxybenzaldehyde (1.01 ml, 8.25 mmol), and CH_2_Cl_2_ (7.0 mL). Chromatography (Telos 40 g, EtOAc in heptane, 0–30 %) gave 0.88 g (89 %) of **37a**: ^**1**^ **H NMR** (CDCl_3_), δ: 8.58 (s, 1H), 7.43 (m, 3H), 7.04–7.14 (m, 1H), 3.89 (s, 3H), 1.23–1.36 (m, 9H) ppm.

**(*R*)‐2‐methyl‐*N*‐(naphthalen‐1‐ylmethylene)propane‐2‐sulfinamide (37b)**: Method C with **36** (0.20 g, 1.6 mmol), PPTS (21 mg, 80 μmol), MgSO_4_ ⋅ H_2_O (0.99 g, 8.2 mmol) and naphthalene‐1‐carbaldehyde (0.45 mL, 3.3 mmol) in CH_2_Cl_2_ (2.6 mL). Chromatography (Telos 20 g, EtOAc in heptane, 0–30 %) gave 0.31 g (72 %) of **37b**: ^**1**^ **H NMR** (CDCl_3_), δ: 9.19 (s, 1H), 9.06 (d, *J*=8.5 Hz, 1H), 8.00–8.14 (m, 2H), 7.90–7.99 (m, 1H), 7.52–7.72 (m, 3H), 1.31–1.40 (m, 9H) ppm.

**(*R*)‐2‐methyl‐*N*‐(quinolin‐4‐ylmethylene)propane‐2‐sulfinamide (37c)**: To a solution of quinoline‐4‐carbaldehyde (0.75 g, 4.8 mmol) and **36** (0.58 g, 4.8 mmol) in anhydrous THF (4.0 mL) under argon was added Ti(OEt)_4_ (1.6 g, 7.2 mmol). The resulting mixture was stirred at rt for 20 h, then quenched with brine (2.0 mL) for 10 min. The resulting suspension was filtered and concentrated under reduced pressure. The filtrate was purified by flash chromatography (Telos 50 g, EtOAc in heptane, 0–85 %) to give 0.47 g (38 %) of **37c**: ^**1**^ **H NMR** (CDCl_3_), δ: 9.23 (s, 1H), 9.11 (d, *J*=4.5 Hz, 1H), 8.78–8.87 (m, 1H), 8.20–8.28 (m, 1H), 7.80–7.89 (m, 2H), 7.72 (ddd, J=8.5, 7.0, 1.4 Hz, 1H), 1.34–1.39 (m, 9H) ppm.

**(*R*)‐*N*‐[(1*S*)‐1‐(3‐methoxyphenyl)propyl]‐2‐methyl‐propane‐2‐sulfinamide (38a)**: Method D with **37 a** (0.17 g, 0.69 mmol), CH_2_Cl_2_ (4.1 mL), and 3 M EtMgBr (0.46 mL). Chromatography (ZIP‐10 g, CH_2_Cl_2_, 100 %, then EtOAc in CH_2_Cl_2_, 0–75 %) gave 0.16 g (86 %) of **38a** as a ∼10 : 1 mixture of diastereomers, which was used without further purification. ^**1**^ **H NMR** (CDCl_3_) δ: 7.23–7.34 (m, 2H), 6.76–6.98 (m, 3H), 4.20–4.36 (m, 1H), 3.74–3.88 (m, 3H), 3.39 (br s, 1H), 1.72–1.94 (m, 2H), 1.14–1.34 (m, 9H), 0.75–0.93 (m, 3H) ppm.

**(*R*)‐2‐methyl‐*N*‐[(*S*)‐1‐(naphthalen‐1‐yl)propyl]propane‐2‐sulfinamide (38b)**: To a cooled (0 °C) solution of sulfinamide **37b** (0.10 g, 0.40 mmol) in toluene (4.0 mL) was added dropwise 3 M EtMgBr (0.27 mL). The resulting mixture was stirred for 1 h, then quenched with a saturated aqueous solution of NH_4_Cl and partitioned between EtOAc and brine. The aqueous phase was further extracted with EtOAc, and the combined organic extracts were dried (Na_2_SO_4_), filtered and concentrated under reduced pressure. The mixture was purified by flash chromatography (ZIP‐10 g, EtOAc in CH_2_Cl_2_ 0–30 %). The residue was taken up in DMSO/MeOH then purified by preparative HPLC to yield 0.12 g (74 %) of **38b** **⋅** **TFA** as a ∼5 : 1 mixture of diastereomers, which was used without further purification. ^**1**^ **H NMR** (CDCl_3_, major diastereomer) δ: 8.18–8.28 (m, 1H), 7.86–7.93 (m, 1H), 7.81 (d, *J*=8.0 Hz, 1H), 7.44–7.61 (m, 4H), 5.15 (t, *J*=6.7 Hz, 1H), 3.47–3.63 (m, 1H), 1.98–2.22 (m, 2H), 1.20 (s, 9H), 0.93 (t, *J*=7.4 Hz, 3H) ppm.

**(*R*)‐2‐methyl‐*N*‐((1*S*)‐1‐(quinolin‐4‐yl)propyl)propane‐2‐sulfinamide (38c)**: Method D with **37c** (0.43 mL, 1.8 mmol), CH_2_Cl_2_ (11.0 mL) and 3 M EtMgBr (1.3 mL). Chromatography (Telos 50 g, EtOAc in heptane, 0–100 %) gave 0.36 g (68 %) of **38c**: ^**1**^ **H NMR** (CDCl_3_) δ: 8.91 (s, 1H), 8.10–8.20 (m, 2H), 7.73 (ddd, *J*=8.4, 6.9, 1.2 Hz, 1H), 7.54–7.65 (m, 1H), 7.42–7.49 (m, 1H), 5.11–5.22 (m, 1H), 3.69 (d, *J*=4.3 Hz, 1H), 2.06–2.17 (m, 2H), 1.22–1.25 (m, 9H), 0.83–0.92 (m, 3H) ppm.

**(1*S*)‐1‐(3‐methoxyphenyl)propan‐1‐amine hydrochloride (39a)**: To a solution of **38a** (0.16 g, 0.59 mmol) in MeOH (0.60 mL), was added HCl (0.30 mL, 4 M in dioxane) under argon. The resulting mixture was stirred at rt for 30 min, after which the mixture was concentrated under reduced pressure. Recrystallisation from MeCN gave 54.4 mg (45 %) of **39a** **⋅** **HCl**: ^**1**^ **H NMR** (CD_3_OD) δ: 7.32–7.44 (m, 1H), 6.93–7.04 (m, 3H), 4.06–4.17 (m, 1H), 3.83 (s, 3H), 1.85–2.10 (m, 2H), 0.89 (t, *J*=7.4 Hz, 3H) ppm.

**(1*S*)‐1‐(quinolin‐4‐yl)propan‐1‐amine (39c)**: To a solution of **38c** (0.36 g, 1.2 mmol) in MeOH (1.0 mL) was added HCl (0.62 mL, 4 M in dioxane). After 30 min, the mixture was concentrated. The residue was triturated with MeCN to yield 0.25 g (79 %) of **39c** **⋅** **2HCl**: ^**1**^ **H NMR** (CD_3_OD) δ: 8.17 (d, *J*=8.5 Hz, 1H), 7.94–8.01 (m, 2H), 7.52–7.69 (m, 3H), 5.19 (t, *J*=7.3 Hz, 1H), 2.07–2.25 (m, 2H), 0.96 (t, *J*=7.4 Hz, 3H) ppm.

**1‐(4‐cyanophenyl)‐5‐oxopyrrolidine‐3‐carboxylic acid (41)**: A microwave vial containing a mixture of 4‐aminobenzonitrile (**12**) (2.0 g, 17 mmol) and 2‐methylenebutanedioic acid (**40**) (2.2 g, 17 mmol) was heated to 160 °C for 1.5 h, then allowed to cool to rt. The resulting gel was sonicated in 2 M NaOH and filtered to remove a pink solid. The filtrate was acidified with 5 M HCl and extracted with EtOAc (x3). The combined organic extracts were dried (Na_2_SO_4_), filtered and concentrated under reduced pressure. The residue was taken up in TFA (10 mL) and stirred at rt for 3 h. The mixture was heated to 55 °C for 18 h, then quenched with a saturated aqueous solution of NaHCO_3_. 2 M NaOH was added and the aqueous phase was extracted using CH_2_Cl_2_, then acidified with 5 M HCl, and extracted with EtOAc (x4). The combined organic extracts were dried (Na_2_SO_4_), filtered and concentrated under reduced pressure. The oily residue was triturated with EtOAc/Et_2_O to produce a sticky solid, which upon trituration with CH_2_Cl_2_ produced a filterable off‐white solid. This was filtered, washed with CH_2_Cl_2_, and dried in vacuo to give 1.3 g (33 %) of **41**: ^**1**^ **H NMR** (DMSO‐*d*_6_) δ: 12.86 (br s, 1H), 7.82–7.91 (m, 4H), 4.05–4.13 (m, 1H), 3.97–4.04 (m, 1H), 3.30–3.43 (m, 1H), 2.79–2.89 (m, 1H), 2.71–2.79 (m, 1H) ppm. **LCMS** *m/z*=229.0 [M−H]^−^.

**(5*R*)‐1‐(4‐carbamimidoylphenyl)‐*N*‐[(1*S*)‐1‐(3‐methoxyphenyl)ethyl]‐5‐oxopyrrolidine‐3‐carboxamide (43)**: Step 1: Method A with racemic **41** (0.15 g, 0.65 mmol), **42** (0.12 g, 0.78 mmol), HATU (0.37 g, 0.98 mmol), DMF (3.0 mL) and *i*‐Pr_2_NEt (0.34 mL, 1.9 mmol). Chromatography (Telos‐12 g, EtOAc in heptane, 0–100 %; then Telos‐25 g, EtOAc in CH_2_Cl_2_, 0–100 %) gave 84 mg (36 %) of the desired diastereomer, which was ultimately identified by its activity against KLK6 after conversion to compound **43**. ^**1**^ **H NMR** (CD_3_OD) δ: 8.58 (d, *J*=7.8 Hz, 1H), 7.75–7.91 (m, 4H), 7.17–7.27 (m, 1H), 6.74–6.93 (m, 3H), 4.90 (p, *J*=7.1 Hz, 1H), 4.06 (t, *J*=9.2 Hz, 1H), 3.93 (dd, *J*=9.8, 5.8 Hz, 1H), 3.75 (s, 3H), 2.81 (dd, *J*=17.2, 9.5 Hz, 1H), 2.60 (dd, *J*=17.2, 6.6 Hz, 1H), (m, 3H), 1.36 (d, *J*=7.0 Hz, 3H) ppm. Step 2: Method B with the above amide (82 mg, 0.23 mmol), NH_2_OH ⋅ HCl (75 mg, 1.1 mmol), *i*‐Pr_2_NEt (0.19 mL, 1.1 mmol), and EtOH (3.4 mL). Then AcOH (3.0 mL) and Ac_2_O (0.11 mL, 1.1 mmol). Then AcOH (3.0 mL) and Zn (0.15 g, 2.3 mmol). Preparative HPLC gave 58 mg (52 %) of **43** **⋅** **TFA**: ^**1**^ **H NMR** (DMSO‐*d*_6_) δ: 9.25 (s, 2H), 9.16 (s, 2H), 8.62 (d, *J*=8.0 Hz, 1H), 7.89–7.94 (m, 2H), 7.82–7.88 (m, 2H), 7.24 (ddd, *J*=8.1, 7.3, 0.7 Hz, 1H), 6.85–6.94 (m, 2H), 6.80 (ddd, *J*=8.2, 2.5, 1.1 Hz, 1H), 4.90 (app p, *J*=7.1 Hz, 1H), 4.09 (dd, *J*=9.9, 8.5 Hz, 1H), 3.96 (dd, *J*=9.9, 5.8 Hz, 1H), 3.75 (s, 3H), 3.29–3.34 (m, 1H), 2.83 (dd, *J*=17.1, 9.5 Hz, 1H), 2.61 (dd, *J*=17.1, 6.7 Hz, 1H), 1.36 (d, *J*=7.0 Hz, 3H) ppm. ^**13**^ **C NMR** (DMSO) δ: 173.6, 171.5, 165.2, 159.8, 159.0 (q, *J*_*C‐F*_=31 Hz), 158.8, 158.5, 146.6, 144.2, 129.9, 129.5, 122.9, 119.0, 118.6, 117.7 (q, *J*_*C‐F*_=300 Hz), 112.4, 112.2, 55.5, 51.0, 48.6, 36.6, 35.8, 23.0 ppm. **HRMS** (ESI) *m/z*: (M+H)^+^ calcd for C_21_H_25_N_4_O_3_^+^: 381.1921; found: 381.1921.

**(3*R*)‐1‐(4‐cyanophenyl)pyrrolidine‐3‐carboxylic acid (46)**: A suspension of **44** (0.26 g, 2.2 mmol), **45** (0.28 g, 2.2 mmol) and Na_2_CO_3_ (804.2 mg, 7.59 mmol) in DMSO (3.0 mL) was heated to 85 °C overnight in a sealed vial. The reaction mixture was diluted with EtOAc and filtered through celite. The combined organic extracts were washed with a saturated aqueous solution of NaHCO_3_ and brine, dried (Na_2_SO_4_) and concentrated under reduced pressure. The crude material was purified by flash column chromatography (Biotage‐10 g, EtOAc in *n*‐heptane, 0–65 %) to give 390 mg (69 %) of the desired ester. To a solution of this ester (0.30 g, 1.3 mmol) in THF (5.0 mL) at 0 °C was added a solution of LiOH ⋅ H_2_O (57 mg, 1.3 mmol) in H_2_O (1.0 mL). The resulting mixture was stirred for 1.5 h, then acidified with 1 M HCl and concentrated under reduced pressure. The residue was extracted with EtOAc (x2), and the combined organic extracts were dried (Na_2_SO_4_), filtered and concentrated under reduced pressure to give 0.22 g (79 %) of **46**: ^**1**^ **H NMR** (CDCl_3_) δ: 7.45–7.53 (m, 2H), 6.48–6.59 (m, 2H), 3.60–3.72 (m, 2H), 3.49–3.56 (m, 1H), 3.38–3.49 (m, 1H), 3.27–3.38 (m, 1H), 2.33–2.45 (m, 2H) ppm.

**(3*R*)‐1‐(4‐carbamimidoylphenyl)‐*N*‐[(1*S*)‐1‐(3‐methoxyphenyl)ethyl]pyrrolidine‐3‐carboxamide (47)**: Step 1: Method A with **46** (80 mg, 0.37 mmol), **42** (60 μL, 0.41 mmol), HATU (0.21 g, 0.55 mmol), DMF (1.5 mL) and *i*‐Pr_2_NEt (0.19 mL, 1.1 mmol). Chromatography (Telos‐10 g, EtOAc in heptane, 0–100 %) gave 120 mg, (91 %) of the desired amide: ^**1**^ **H NMR** (DMSO‐*d*6) δ: 8.48 (d, *J*=8.03 Hz, 1H), 7.57–7.48 (m, 2H), 7.28–7.13 (m, 1H), 6.91–6.85 (m, 2H), 6.79 (ddd, *J*=8.2, 2.4, 1.1 Hz, 1H), 6.62–6.56 (m, 2H), 4.95–4.84 (m, 1H), 3.77–3.72 (m, 3H), 3.55–3.47 (m, 1H), 3.44–3.35 (m, 2H), 3.30–3.25 (m, 1H), 3.16 (p, *J*=7.5 Hz, 1H), 2.21 (dtd, *J*=12.1, 7.3, 7.3, 4.6 Hz, 1H), 2.10–2.00 (m, 1H), 1.35 (d, *J*=7.0 Hz, 3H) ppm. Step 2: Method B with the above amide (60 mg, 0.17 mmol), NH_2_OH ⋅ HCl (57 mg, 0.82 mmol), *i*‐Pr_2_NEt (0.14 mL, 0.82 mmol) and EtOH (2.9 mL). Then AcOH (2.0 mL) and Ac_2_O (80 μL, 0.86 mmol). Then AcOH (2.0 mL) and Zn (0.11 g, 1.7 mmol). Preparative HPLC gave 35 mg (42 %) of **47** **⋅** **TFA**: ^**1**^ **H NMR** (DMSO‐*d*6) δ: 8.82 (s, 2H), 8.50 (d, *J*=8.0 Hz, 1H), 8.39 (s, 2H), 7.72 (d, *J*=9.0 Hz, 2H), 7.21–7.28 (m, 1H), 6.86–6.91 (m, 2H), 6.80 (ddd, *J*=8.2, 2.6, 1.0 Hz, 1H), 6.66 (d, *J*=9.0 Hz, 2H), 4.90 (p, *J*=7.2 Hz, 1H), 3.52–3.60 (m, 1H), 3.30–3.49 (m, 3H), 3.18 (dt, *J*=14.9, 7.6 Hz, 1H), 2.18–2.29 (m, 1H), 1.99–2.11 (m, 1H), 1.35 (d, *J*=7.0 Hz, 3H) ppm. **LCMS** *m/z*=367.2 [M+H]^+^.

**4‐((*S*)‐5‐((((*S*)‐1‐(3‐methoxyphenyl)ethyl)amino)methyl)‐2‐oxooxazolidin‐3‐yl)benzonitrile (48)**: To a solution of **17** (100 mg, 0.46 mmol) in THF (1.1 mL) was added Et_3_N (0.08 ml, 0.6 mmol) followed by methanesulfonyl chloride (0.05 ml, 0.6 mmol). A suspension resulted which was stirred at rt for 16 h. The resulting mixture was quenched with H_2_O and concentrated. The residue was taken up in CH_2_Cl_2_ and passed through a hydrophobic phase separator, further rinsing with CH_2_Cl_2_. The filtrate was concentrated under reduced pressure. The residue was taken up in DMF (0.5 mL) and transferred to a microwave vial containing **42** (104 mg, 0.69 mmol). The resulting mixture was subjected to microwave irradiation at 120 °C for 60 min and then 130 °C for 90 min. Thermal heating was then used at 100 °C for 16 h. The resulting mixture was partitioned between EtOAc and brine/water. The aqueous phase was further extracted with EtOAc and the combined organic extracts washed with brine (x2), dried (Na_2_SO_4_), filtered and concentrated, and purified by column chromatography (Telos 12 g, EtOAc in DCM, 0–80 %), then (SNAP Ultra‐10 g, EtOAc in DCM, 0–50 % over 12 CV then up to 65 %) to give 80 mg of **48**, which taken on directly in the next step.

**4‐((*S*)‐5‐((((*S*)‐1‐(3‐methoxyphenyl)ethyl)amino)methyl)‐2‐oxooxazolidin‐3‐yl)benzimidamide (49)**: Method B with **48** (159 mg, 0.45 mmol), NH_2_OH ⋅ HCl (150.9 mg, 2.17 mmol), *i*‐Pr_2_NEt (0.38 ml, 2.17 mmol) in EtOH (7.8 mL). Then AcOH (5.2 mL) and Ac_2_O (0.21 ml, 2.26 mmol). Then AcOH (5.2 mL) and Zn (592 mg, 9.05 mmol). Preparative HPLC gave 144.5 mg (66 %) of **49** **⋅** **TFA**: ^**1**^ **H NMR** (CD_3_OD) δ: 7.74–7.90 (m, 4H) 7.40 (t, *J*=7.9 Hz, 1H) 6.95–7.14 (m, 3H) 5.02–5.14 (m, 1H) 4.48 (q, *J*=6.9 Hz, 1H) 4.30 (t, *J*=9.2 Hz, 1H) 3.79–3.89 (m, 4H) 3.40 (dd, *J*=13.5, 2.5 Hz, 1H) 3.22 (dd, *J*=13.5, 10.0 Hz, 1H) 1.72 (d, *J*=6.8 Hz, 3H) ppm. **LCMS** *m/z*=369.2 [M+H]^+^.

**Enzyme inhibition assays**: All chemicals were purchased from Sigma Aldrich (Gillingham, UK) unless otherwise stated. All experiments were conducted in 384 well black, low volume microplates (Greiner 784076) with a final volume of 20 μL and compounds were dispensed into assay plates using an ECHO 550 acoustic dispenser (Labcyte). KLK6 (5 nM) (prepared as in ref. 21) was incubated with test compounds for 30 min at room temperature in assay buffer (50 mM Tris, pH 7.5, 150 mM NaCl, 1 mM EDTA, 0.05 % tween 20, 0.1 % bovine serum albumin (BSA), 1 mM dithiothreitol (DTT)) before the reaction was started by addition of 20 μM Boc‐Phe‐Ser‐Arg‐AMC substrate (Bachem). Fluorescence intensity was read at ex/em 355/460 following incubation for 2 h in the dark at room temperature.

Assays using KLK7 and KLK8 (2B scientific) were conducted in the same manner as for KLK6 with the following exceptions. Both KLK7 and KLK8 required activation before assaying. KLK7 was combined with an equal volume of 20 μg/ml thermolysin (R&D systems) in activation buffer (50 mM Tris, pH 7.5, 10 mM CaCl_2_, 150 mM NaCl and 0.05 % Brij‐L23) and incubated at 37 °C for 2 h, the reaction was stopped with the addition of 100 mM EDTA in stop solution (50 mM Tris, 150 mM NaCl, pH 8.5). KLK8 was combined with an equal volume of 0.4 mU/mL lysyl endopeptidase (Alpha laboratories) in activation buffer (50 mM Tris, pH 8.0) and incubated at 37 °C for 30 min. Activated KLK7 (40 nM) and KLK8 (25 nM) were assayed with 40 μM Mca‐RPKPVE‐Nval‐WRK(Dnp)‐NH2 fluorogenic MMP substrate (R&D systems) and 25 μM Boc‐V‐P‐R‐AMC substrate (R&D systems), respectively.

Human thrombin was purchased from Biopur. Human trypsin was purchased from Polymun Scientific. Human Factor Xa was purchased from Enzo Life Sciences. Thrombin (20 nM), trypsin (0.000015 %) or Factor Xa (10 nM) were assayed in a similar manner to the KLKs but were incubated with compound for 20 min at room temperature in assay buffer (50 mM Tris HCl, pH 7.6, 100 mM NaCl, 1 mg/ml CHAPS, 0.05 % bovine serum albumin (BSA), 1 mM dithiothreitol (DTT)) before the addition of 5 μM, 2.5 μM or 1.5 μM Rhodamine 110 substrate (Cambridge bioscience ltd) respectively. Fluorescence intensity was then read at ex/em 485/535 following incubation for 1 h at room temperature (thrombin and factor Xa) or 20 min (trypsin).

**Kinetic aqueous solubility**: The aqueous solubility of the test compounds was measured using laser Nephelometry. Compounds were subjected to serial dilution from 10 mM to 0.5 mM in DMSO. An aliquot was then mixed with Milli‐Q water to obtain an aqueous dilution plate with a final concentration range of 250–12 μM, with a final DMSO concentration of 2.5 %. Triplicate aliquots were transferred to a flat bottomed polystyrene plate which was immediately read on the NEPHELOstar (BMG Lab Technologies). The amount of laser scatter caused by insoluble particulates (relative nephelometry units, RNU) was plotted against compound concentration using a segmental regression fit, with the point of inflection being quoted as the compounds aqueous solubility (μM).

**Thermodynamic solubility**: Test compound (3 mg) is accurately weighed into a Micronics tube. Extra tubes for each control compound, Warfarin (high solubility control) and Cinnarizine (low solubility control) were also accurately weighed. To each tube is added 1 mL of Milli‐Q water. All samples are vortexed for 20 s and placed in a bench top incubator shaker set at 1300 rpm at 37 °C for 5 h. After the incubation, samples are centrifuged at 10,000 rpm for 3 min. 100 μL aliquots of the supernatant in duplicate were placed in a 96‐well plate. All samples, calibration standards and QC samples are analyzed using a validated HPLC method on the Dionex Ultramate 3000 HPLC system. The concentration determined from the supernatant was then reported as the solubility of each compound in μg/mL.

**Microsomal Intrinsic clearance (CLint) assay**: Test compound (0.5 μM) was incubated with female CD1 mouse liver microsomes (Xenotech LLC TM; 0.5 mg/mL 50 mM potassium phosphate buffer, pH 7.4) and the reaction started with addition of excess NADPH (8 mg/mL 50 mM potassium phosphate buffer, pH 7.4). Immediately, at time zero, then at 3, 6, 9, 15 and 30 min an aliquot (50 μL) of the incubation mixture was removed and mixed with acetonitrile (100 μL) to stop the reaction. Internal standard was added to all samples, the samples centrifuged to sediment precipitated protein and the plates then sealed prior to UPLC‐MS/MS analysis using a Quattro Premier XE (Waters Corporation, USA). XLfit (IDBS, UK) was used to calculate the exponential decay and consequently the rate constant (*k*) from the ratio of peak area of test compound to internal standard at each timepoint. The rate of intrinsic clearance (CLint) of each test compound was then calculated using the following calculation: CLint (mL/min/g liver)=*k*×V×Microsomal protein yield. Where V (mL/mg protein) is the incubation volume/mg protein added and microsomal protein yield is taken as 52.5 mg protein/g liver. Verapamil (0.5 μM) was used as a positive control to confirm acceptable assay performance.

**Hepatocytes Intrinsic clearance (CLint) assay**: Cryopreserved vials of mouse cryopreserved hepatocytes, supplied by Life Technologies, were thawed according to manufacturer's instructions and cells re‐suspended in Williams Medium E (WME) containing cell maintenance supplement pack (CM4000, Life Technologies). Hepatocytes were incubated in suspension (0.5x10^6^ cells/mL) in 48 well non‐collagen coated cell culture plates for 10 min at 37 °C, 95 % O_2_ 5 % CO_2_. Upon addition of an equal volume of supplemented WME containing 1 μM test compound, an aliquot of incubation solution was removed to acetonitrile containing internal standard (final concentration 0.5 μM test compound and a cell density of 0.25×10^6^ cells/mL). Similarly, aliquots were removed at 3, 6, 9, 15, 30, 45, 60, 90 and 120 min. 100 μL of 80 : 20 H_2_O/MeCN was added to all samples and the analysis plate was centrifuged for 10 min at room temperature prior to injection and analysis of samples by UPLC‐MS/MS. The response (area ratio of test compound to internal standard) was plotted against time using an exponential decay model and rate of disappearance calculated. Hepatocyte CLint (mL/min/10^6^ cells) was scaled to in vivo CLint (mL/min/g liver) using the hepatocellularity scaling factor of 120×10^6^ cells/g of liver.

**Fluorescence‐based CYP Inhibition using recombinantly expressed CYP bactosomes**: Fluorescence CYP inhibition studies were conducted at 37 °C in 96‐well, flat‐bottom, clear polystyrene plates. Incubation mixtures containing EasyCYP bactosomes (1000 pmol/mL, 10 mg/mL Cypex TM), fluorigenic substrate (Cypex TM) and 50 mM potassium phosphate buffer (pH 7.4) were prepared at the following final concentrations: CYP1A2, 5 pmol/mL+0.5 μM Ethoxyresorufin (ER); CYP2 C9, 10 pmol/mL+30 μM 7‐methoxy‐4‐(trifluoromethyl)‐coumarin (MFC); CYP2C19, 5pmol/mL+25 μM 3‐Cyano‐7‐Ethoxycoumarin (CEC); CYP2D6, 10 pmol/mL+6 μM 7‐methoxy‐4‐(aminomethyl)‐coumarin (MAMC); CYP3A4, 10 pmol/mL+1 μM Diethoxyfluorescein (DEF) & 10 pmol/mL+15 μM 7‐Benzyloxyquinoline (BQ). Bactosome control protein was included in reactions to give a final concentration of 0.025 mg/mL. Reaction times were verified to be within the limits of kinetics linearity. For each isoform, 220 μL of incubation mix was added to each well of a 96‐well, flat‐bottom, clear polystyrene plate. Test compounds were prepared as 5 mM solutions in DMSO and serially diluted 1 in 3.03, 1 in 3.3 alternatively in a v‐bottomed 96 well plate to give a concentration range of 5, 1.65, 0.5, 0.165, 0.05, 0.0165, 0.05 mM. Positive control inhibitor, Miconazole, was prepared as a 0.5 mM solution in DMSO and similarly diluted. 5 μL of each concentration was mixed with 220 μL of the incubation mix and pre‐incubated at 37 °C for 5 min (final test compound concentration range: 100, 33, 10, 3.3, 1.0, 0.33, 0.1 or 10, 3.3, 1.0, 0.33, 0.1, 0.033, 0.01 and 0 μM, Miconazole 10, 3.3, 1.0, 0.33, 0.1, 0.033, 0.01 and 0 μM). Reactions were initiated by the addition of 25 μL regenerating cofactor solution (23 mM Glucose‐6‐Phosphate, 2.2 mM NADP, 6 Units per ml Glucose‐6‐Phosphate Dehydrogenase (from Baker's yeast S. cerevisiae) in 2 % w/v NaHCO_3_, Sigma) and subsequent production of fluorescence metabolite measured at 1 min intervals over a 10 min period using a BMG Optima fluorescence detector (ER: Exc 530 nm, Em 590 nm, MFC: Exc 410 nm, Em 530 nm, CEC: Exc 410 nm, Em 530 nm, MAMC: Exc 405 nm, Em 460 nm, DEF: Exc 405 nm, Em 560 nm, 7BQ: Exc 485 nm, Em 530 nm). Fluorescence responses were adjusted to a percentage of uninhibited control before plotting against compound concentration and deriving the IC_50_ by fitting to a 4 parameter logistic regression model.

**Plasma protein binding assay**: Briefly, a 96 well equilibrium dialysis apparatus was used to determine the free fraction in plasma for each compound (HT Dialysis LLC, Gales Ferry, CT). Membranes (12–14 kDa cut‐off) were conditioned in deionized water for 60 minutes, followed by conditioning in 80 : 20 deionized water: ethanol for overnight at 4 °C and then rinsed in isotonic buffer before use. Female CD1 mouse plasma was removed from the freezer and allowed to thaw on the day of experiment. Thawed plasma was then centrifuged (Allegra X12‐R, Beckman Coulter, USA), spiked with test compound (final concentration 10 μg/mL), and 150 μL aliquots (n=6 replicate determinations) loaded into the 96‐well equilibrium dialysis plate. Dialysis vs isotonic buffer (150 μL) was carried out for 5 h in a temperature‐controlled incubator at 37 °C (Barworld scientific Ltd, UK) using an orbital microplate shaker at 100 rpm (Barworld scientific Ltd, UK). At the end of the incubation period, 50 μL aliquots of plasma or buffer were transferred to micronic tubes (Micronic B.V., the Netherlands) and the composition in each tube balanced with control fluid (50 μL), such that the volume of buffer to plasma is the same. Sample extraction was performed by the addition of 200 μL of acetonitrile containing an appropriate internal standard. Samples were allowed to mix for 1 min and then centrifuged at 3000 rpm in 96‐well blocks for 15 min (Allegra X12‐R, Beckman Coulter, USA) after which 150 μL of supernatant was removed to 50 μL of water. All samples were analyzed by UPLC‐MS/MS on a Quattro Premier XE Mass Spectrometer (Waters Corporation, USA). The unbound fraction was determined as the ratio of the peak area in buffer to that in plasma.

**Plasma stability assay**: Test compound (10 μM) was incubated in pre‐warmed plasma at 37 °C (that is buffered to pH 7.4 in ratio of 70 : 30 plasma to buffer). Immediately, at time zero, then at 30, 60, 120, and 180 min, a 50 μL aliquot of the incubation mixture was removed and mixed with 200 μL acetonitrile containing Donepezil as the internal standard (50 ng/mL) to stop the reaction. The samples were centrifuged to sediment the precipitated protein and the plates then sealed prior to UPLC‐MS/MS analysis using a Quattro Premier XE (Waters Corporation, USA). XLfit (IDBS, UK) was used to calculate the exponential decay and consequently the rate constant (k) from the ratio of peak area of test compound to internal standard at each time point. The half‐life was calculated for each test compound from the rate by using the following calculation: t_1/2_=0.693/k

**Invasion assay**: The HCT116 colorectal adenocarcinoma cell line (ATCC® CCL‐247™) was maintained in Dulbecco's Modified Eagle Medium (DMEM) with 4.5 mg/L glucose, l‐glutamine w/o sodium pyruvate, supplemented with 10 % FBS and 1 % penicillin/streptomycin. HCT116 cell invasion was performed in 24‐well chambers with Matrigel coated inserts with a 0.8 μm pore size (Corning Inc., Corning, NY). In the lower chamber of each well 0.5 mL of media supplemented with 10 % FBS was added as a chemoattractant. HCT116 cell suspension was prepared at a concentration of 0.5×10^6^ cells/mL in serum‐free medium. Cells in suspension were pre‐treated with different concentrations of test compounds for 20 min before seeding onto inserts. 200 μL of cell suspensions (1×10^5^ cells) were plated into each insert with six inserts plated for each condition. The cells were allowed to invade for 24 h in the incubator at 37 °C in 5 % CO_2_. After incubation, the media was removed and inserts were rinsed briefly with 1 X PBS and swabbed gently inside with a cotton tip to remove non‐invading cells. The invading cells outside the insert were fixed in 100 % methanol for 2 min, stained with 1 % toluidine blue in 1 % borax for 2 min, rinsed twice in ddH_2_O, swabbed gently again inside with a cotton tip and air‐dried. The stained membranes were cut from the inserts and the incorporated toluidine blue was dissolved in 200 μL of 0.1 M citric acid in a 96 well plate while shaking for 5 min on a high‐speed titer plate shaker. The samples were transferred to a new 96 well plate and read at 560 nm on a Synergy 2 Multi‐Detection Microplate Reader (Bio‐Tek Instruments, Inc., Winooski, VT).

## Conflict of interest

The authors declare no conflict of interest.
